# Supplementary material for: Dominant ARF3 variants disrupt Golgi integrity and cause a neurodevelopmental disorder recapitulated in zebrafish
Source: Nat Commun. 2022 Nov 11;13:6841. doi: 10.1038/s41467-022-34354-x (PMC9652361; doi:10.1038/s41467-022-34354-x)
Supplement: Supplementary file 1 — Supplementary Information [file 41467_2022_34354_MOESM1_ESM.pdf]

Dominant ARF3 variants disrupt Golgi integrity and cause a neurodevelopmental disorder recapitulated in zebrafish

Fasano, Muto, Radio, *et al.*

SUPPLEMENTARY INFORMATION

Supplementary clinical reports

Supplementary methods

Supplementary tables

Sup. Table 1. Clinical features of the five subjects with pathogenic *ARF3* missense mutations

Sup. Table 2. List of the *ARF3* missense mutations identified in the study.

Sup. Tables 3-7. WES statistics and data output (subjects 1 to 5).

Sup. Table 8. Pathogenic variants in ARF3 paralogs, and HRAS, NRAS and KRAS GTPases affecting codons corresponding and/or adjacent to the residues mutated in ARF3.

Sup. Table 9. ARF3-GTP hydrogen bonds present for more than 50% of simulation time in the ARF3:COPG1-COPZ1 complex considering $ARF3^{WT}$, $ARF3^{D67V}$ and $ARF3^{P47S}$.

Sup. Table 10. ARF3:COPG1 hydrogen bonds present for more than 40% of simulation time in the ARF3:COPG1-COPZ1 complex considering $ARF3^{WT}$, $ARF3^{D67V}$ and $ARF3^{P47S}$.

Sup. Table 11. Primers list

Sup. Table 12. Overview of the statistical analyses.

Supplementary Figure 1. Clinical features of individuals with *de novo ARF3* mutations.

Supplementary Figure 2. ARF3 multiple sequence alignment and mutations tolerance landscape.

Supplementary Figure 3. 2D projection of $ARF3^{WT}$, $ARF3^{D67V}$ and $ARF3^{P47S}$ on the respective essential subspace along eigenvectors 1 and 2 resulting from MD simulations.

Supplementary Figure 4. Myc-tagged ARF3 protein levels in COS-1 cells and zebrafish embryos during gastrulation and segmentation stages.

Supplementary Figure 5. RT-PCR of myc-tagged $ARF3^{WT}$, $ARF3^{L12V/D67V}$ and $ARF3^{K127E}$ and quantification of total fluorescent intensity of mCherry tagged-$ARF3^{K127E}$, $ARF3^{L12V/D67V}$ and $ARF3^{D93N}$ upon overexpression in COS-1 cells.

Supplementary Figure 6. Nucleotide exchange and GTP hydrolysis measurements of $ARF3^{WT}$, $ARF3^{K127E}$, $ARF3^{L12V/D67V}$, $ARF3^{D93N}$ and $ARF3^{T32N}$.

Supplementary Figure 7. 3D rendering of Golgi morphotypes observed in cells expressing $ARF3^{WT}$, $ARF3^{Q71L}$ and $ARF3^{T31N}$, relative to Figure 3.

Supplementary Figure 8. Ultrastructural morphology of Golgi apparatus in COS-1 cells transfected with $ARF3^{WT}$, $ARF3^{K127E}$ and $ARF3^{D93N}$ revealed by TEM imaging.

Supplementary Figure 9. Endogenous mRNA expression of *arf3a* and *arf3b* paralogs during early zebrafish embryogenesis.

Supplementary Figure 10. Quantification of *trans*-Golgi morphology in precursor cells of the animal pole of 6 hpf zebrafish embryos expressing $ARF3^{WT}$, $ARF3^{K127E}$ and $ARF3^{D93N}$.

Supplementary figure 11. Intracellular Tfn distribution after 5 and 30 minutes of incubation.

Supplementary Figure 12. Co-localization analysis of Tfn and Rab11 at PN upon 30 min of Tfn incubation.

Supplementary Figure 13. Altered lysosomal trafficking of Tfn upon 30 min of incubation in cells expressing ARF3 mutations.

Supplementary Figure 14. Expression of myc-tagged ARF3 during early zebrafish embryogenesis.

Supplementary Figure 15. Incidence of gross phenotypes in fish overexpressing WT and mutant ARF3 at 48 hpf and survival curve of *arf3a* and *arf3b* MO-injected fish at 24 and 48 hpf.

Supplementary Figure 16. Anterior brain volume at 48 hpf is reduced in zebrafish expressing $ARF3^{K127E}$.

Supplementary figure 17. Zebrafish embryos expressing ARF3 mutants exhibit thinning of the major tract of the forebrain white matter.

Supplementary Figure 18. Ectopic proliferative cells are found within the developing diencephalon of zebrafish expressing $ARF3^{K127E}$ and $ARF3^{D93N}$ mutants.

Supplementary Figure 19. Impaired spindle morphology of mitotic cells within the developing forebrain of zebrafish embryos expressing $ARF3^{K127E}$ and $ARF3^{D93N}$.

Supplementary Figure 20. Assessment of the number of somites in ARF3 mutants at 15 hpf.

Supplementary Figure 21. Overexpression of $ARF3^{K127E}$ and $ARF3^{D93N}$ in zebrafish embryos impairs convergence and extension movements during gastrulation.

Supplementary Figure 22. Comparison of the FASTA sequences available in the UniProt database and the PDB entry 3TJZ.

Supplementary References

Raw uncropped blots and gels relative to supplementary figures

Supplementary Clinical Reports

<u>*Subject 1*</u>

The proband, a female, is the 2nd child of healthy non-consanguineous Italian parents. Family history was unremarkable. She was born at term of a pregnancy complicated by maternal diabetes by cesarean section. Birth weight was 2,700g (10th-25th centile, -1.42 SD), length 46 cm (3th centile, -2.05 SD) and OFC 33.5 cm (50th centile, -1.11 SD). Apgar scores were 6 and 7 at 1 and 5 minutes, respectively. She required resuscitation due to respiratory distress and cyanosis. Severe central hypotonia and peripheral hypertonia were noted early at birth. Motor developmental milestones were significantly delayed. Feeding and swallowing difficulties were present.
Clinical evaluation at 5 months disclosed severe neurodevelopmental delay, microsomia (weight 3,770 g, << 3rd centile, -5.95 SD; length 52 cm, << 3rd centile, -6.6 SD; OFC 37cm, << 3rd centile, -4.56 SD), seizures (onset 2 months), inguinal hernia, congenital heart defect (pulmonary anomalous venous return and ostium secundum atrial septal defect), moderate unilateral pyelectasis and minor skeletal defects (*i.e.,* 11 rib pairs, scoliosis). The craniofacial appearance was characterized by bitemporal narrowing, hypotelorism, long eyelashes and flat nasal bridge with small nose (**Supplementary Figure 1**), principally related with microcephaly.
Serial brain MRIs (1 month - 3 years) showed a progressive severe and diffuse cortical atrophy, lateral ventricular enlargement, severe corpus callosum and brainstem hypoplasia particularly affecting the pons, cerebellar inferior vermis hypoplasia.
At 5 months of age, the EEG analysis revealed a disorganized pattern characterized by diffuse multifocal epileptic discharges. Episodes of hypertonus and dystonia, without a corresponding EEG change were observed. The treatment included phenobarbital (5 mg/Kg/die), baclofen (0.6 mg/Kg/die), lorazepam (2 mg/Kg/die), morphine and clonidine. Biochemical and metabolic screening as well as postnatal genetic analyses including array-CGH were negative.

<u>*Subject 2*</u>

The proband, a male, is the 4th child of healthy non-consanguineous French parents. Family history was unremarkable. He was born at term of an uneventful pregnancy by vaginal delivery. Birth weight was 2,800g (10th-25th centile, -1.24 SD), length 46.5cm (3-10th centile, -1.73 SD) and OFC 31.5 cm (below the first centile, -2.33 SD). Apgar scores were 10 at both 1 and 5 minutes. First two weeks of life were considered as normal. Severe central hypotonia and distal hypertonus with opisthotonos episodes were noted at the age of 1 month. Motor milestones were significantly delayed (no

independent sitting and no standing). Feeding and swallowing difficulties required G-tube feeding. Clinical evaluation at 3 months disclosed severe neurodevelopmental delay, microsomia (weight 4.800 g, << 3rd centile, -2.38 SD; length 55 cm, << 3rd centile, -3.17 SD; OFC 37 cm, << 3rd centile, -3.02 SD). Recurrent dystonic episodes were noted. No dysmorphic features other than those associated with microcephaly were observed. Serial brain MRIs (3 month - 14 months) showed a progressive extremely severe and diffuse cortical atrophy, lateral ventricular enlargement, progressive pontocerebellar hypoplasia and a thin corpus callosum without involvement of the cerebellar vermis.

Convergent strabismus with normal fundus examination was noted at age 2 years. Heart and abdominal ultrasounds were found to be normal at age 3 years. At last evaluation (3 years old), developmental delay was severe as the patient is not able to speak neither to walk. Spasticity requiring Baclofen iterative injections was a main concern to the parents.

Biochemical and metabolic screening as well as postnatal genetic analyses including SNP array were negative.

<u>Subject 3</u>

The proband, a girl, is the only child of healthy non-consanguineous Italian parents. Family history was unremarkable except for a single febrile seizure in the father. The girl was born at term by vaginal delivery, after an uneventful pregnancy. Birth weight was 3,240 g (> 25th centile, -0.73 SD). Apgar scores were 8 - 9. No dysmorphic features were noticeable, but neonatal jaundice and a fracture of the right clavicle were observed. The girl acquired head control at 2 months, the sitting position at 5 months, and autonomous walking at 3 years 9 months.

Between 8 and 26 months, she experienced six febrile seizures, most of which were successfully stopped with intrarectal Diazepam. Multiple interictal EEGs were normal. Brain MRI scan at 17 months showed thin corpus callosum, severe hypomyelination, moderate ventricular dilatation and severe hypoplasia of the anterior part of the temporal lobes.

Clinical evaluation at 30 months revealed developmental delay with a head circumference of 46 cm (-1.44 SD). At last evaluation, at 3 years 10 months, the girl could only pronounce a few words. Her head circumference and height were 49.4 cm (-1.01 SD) and 101 cm (-0.02 SD), respectively. Plasma and urine metabolic workout were unremarkable, as well as karyotype, array-CGH, and targeted sequencing of a panel of epilepsy-associated genes. Echocardiography and chest X-ray were normal.

Subject 4

The proband, a girl, is the first child of healthy non-consanguineous Italian parents. Family history was unremarkable. She was born at term of a pregnancy complicated by uterine contractions. Birth weight was 3,600 g (+0.73 SD), length 50.5 cm (+0.42 SD) and OFC 33 cm (-0.99 SD). Apgar score was 10 at 1 and 5 minutes. Neonatal discharge was normal. Mild hypotonia was noted early. Motor developmental milestones were significantly delayed.
Clinical evaluation at 1 year disclosed developmental delay and normal growth with microcephaly (OFC 42.3 cm, < 3rd centile, -2.33 SD). The craniofacial appearance was characterized by brachycephaly, deep-set eyes, epicanthal folds, short philtrum, anteverted ears (**Supplementary Figure 1**). Moreover, a mild pectus excavatum and a café-au-lait spot on the leg were evident. Follow up at 3, 5 and 7 years didn't disclose additional features or medical problems. A brain MRIs (1 year) showed microcephaly and thin corpus callosum. Biochemical screening as well as postnatal genetic analyses, including *FMR1*, CGH array, NGS panel for Angelman/Rett syndrome, *UBE3A* MLPA and NGS panel for ID (700 genes) were negative.

Subject 5

The child is the 2nd son of healthy unrelated parents. He has a healthy sister (older) and a healthy brother (younger). Family history is unremarkable. Pregnancy was uneventful; the mother performed ultrasound examinations which gave normal results. Delivery was at term with normal auxologic data. APGAR score was 9 and 10 at 1 and 5 minutes, respectively. Neonatal period was normal. We evaluated the child at 12 years of age. His growth was always normal for weight, height and head circumference with values between 50th and 75th centile. No major malformation was evident. Achievement of the common pediatric milestones was delayed: he could seat at 12 months, walk independently at 34 months, say his first word at 36 months of age. He underwent speech therapy, augmentative, alternative communication and occupational therapy. Hearing and vision were normal. No seizures have been observed. Brain MRI showed a thin corpus callosum in its medium and posterior portions, mega cisterna magna, and severe hypomyelination. CGH array and Fragile-X analyses and mutation scan using a panel of genes related to neurodevelopmental abnormalities were normal. No facial dysmorphisms were evident. His skin was quite dry (cutaneous xerosis). A left dorsal hump was noticed.

Supplementary Methods

All the reported software and tools for human sequences analysis are open-access or publicly available and all the software used in this study are included here and in the Reporting summary.

Sequencing

The commercial software Illumina Experiment v.1.19.1 was used to create sample sheets for sequencing, along with the Illumina BaseSpace Hub v.5.43 for fastq file generation (https://help.basespace.illumina.com/overview/software-overview).

WES and variant calling, annotation, filtering and prioritization

Subject 1:

DNA of the affected subject and his parents was extracted from leukocytes. Exome capture was carried out using Sure Select QXT Human All Exon V7 (Agilent), and sequencing was performed on a NovaSeq6000 platform (Illumina). Raw data were processed and analyzed using an in-house implemented pipeline previously described (Bauer *et al*., 2018; Motta *et al*., 2020; Radio *et al*., 2021), which is based on the GATK Best Practices (Van der Auwera *et al*., 2013). The UCSC GRCh37/hg19 version of genome assembly was used as a reference for reads alignment by means of BWA-MEM v.2 (Li and Durbin, 2009, Li, 2013) tool and subsequent variant calling with HaplotypeCaller (GATK v3.8). SnpEff v.5.0 (Cingolani *et al.,* 2015) and dbNSFP v.4.2 (Liu *et al.,* 2013) tools were used for variants annotation. Combined Annotation Dependent Depletion (CADD) v.1.64 (Kircher *et al.,* 2014), Mendelian Clinically Applicable Pathogenicity (M-CAP) v.1.0 (Jagadeesh *et al.,* 2016) and Intervar v.2.0.1 (Li and Wang, 2017) were considered for functional impact prediction. Variant filtering was performed to consider variants affecting either coding sequences or splice site regions. High-quality variants were filtered against public (dbSNP150 and gnomAD V.2.1.1, MAF threshold ≤ 0.1% or unknown frequency) or in-house (~2,800 population-matched exomes, MAF threshold <1%) databases. Among 105,025 high-quality variants, 16,273 were predicted to be functionally relevant, with 453 representing rare/private variants. A further stratification taking in account genotype, inheritance model, and predicted functional impact, indicated the *de novo* c.379A>G substitution (p.Lys127Glu) in *ARF3* as the best candidate event underlying the trait. For sequencing statistics and data output see **Supplementary Table 3**. Validation and segregation of the variant in *ARF3* were assessed by Sanger sequencing.

Subject 2:

DNA of the affected subject and his parents was extracted from leukocytes. Exome capture was carried out using Sure Select XT Clinical Research Exome (Agilent) and sequencing was performed on an Illumina HiSeq4000 platform. Base calling is performed using the Real-Time Analysis software sequence pipeline (2.7.6) with default parameters. Sequence reads were mapped to the human genome build (GRCh37/hg19) using Elandv2e (Illumina, CASAVA 1.8.2) allowing multiseed and gapped alignments. Variant filtering was performed to consider variants affecting either coding sequences or splice site regions. High-quality variants were filtered against public (dbSNP150 and gnomAD V.2.1.1, MAF threshold ≤ 0.01% or unknown frequency for dominant and X-linked transmission and <0.1% for recessive transmission). After stratification taking into account genotype, inheritance model, and predicted functional impact, indicated two *de novo* c.34C>G and c.200A>T missense substitutions (p.Leu12Val and p.Asp67Val) in *ARF3* as the best candidate gene underlying the trait. For sequencing statistics and data output see **Supplementary Table 4**. Cloning was used to determine that both variants were on the same allele. Validation and segregation of the variants in *ARF3* were assessed by Sanger sequencing.

Subject 3:

We performed trio-based whole exome sequencing (WES) in the patient and both her parents. We used the Human Core Exome Kit + RefSeq V1 (Twist Bioscience) for target enrichment, and sequenced the DNA libraries by a paired-end 2x150 bp protocol on the NextSeq550 (Illumina, San Diego, CA USA). Data analysis was carried out as previously reported (Vetro *et al.,* 2021). Briefly, we aligned the sequencing reads to the GRCh37/hg19 human genome reference assembly by the BWA software package (Li and Durbin, 2009, Li, 2013) and then used the GATK suite for base quality score recalibration, realignment of insertion/deletions (InDels), and variant calling, according to GATK Best Practices recommendations (DePristo *et al.,* 2011). For sequencing statistics and data output see **Supplementary Table 5**. We annotated and filtered exonic/splice-site single-nucleotide variants (SNVs) and coding InDels by the VarSeq software (Golden Helix, Inc v1.4.6, Bozeman, MT). We considered different inheritance patterns, focusing on variants with minor allele frequency (MAF) lower than 0.01 in the GnomAD database and evaluated the potential functional impact of SNVs and InDels by the pre-computed genomic variants score from dbNSFP, which was integrated in the annotation pipeline (Liu *et al.,* 2011), and by the tools Combined Annotation Dependent Depletion (CADD) v.1.4 (http://cadd.gs.washington.edu/) and GERP++ (Cooper *et al.,* 2005). For selected variants, we visually inspected the quality of reads alignment using the Integrative Genomics Viewer (IGV v2.4) (Thorvaldsdóttir *et al.,* 2013).

Variant filtering and prioritization took into account population allele frequency, inheritance model, and predicted functional impact. The *de novo* c.139C>T (p.Pro47Ser) substitution in *ARF3* was validated by Sanger sequencing.

Subject 4 and Subject 5:

Trio-based WES was performed in both families. Genomic DNA was extracted from peripheral blood samples using standard procedures. Coding exons and flanking intronic regions were captured using the Clinical Research Exome v.2 kit (Agilent Technologies, Santa Clara, CA). Sequencing was performed using a NextSeq500 Illumina system with 150 bp paired-end reads. Reads were aligned to human genome build GRCh37/hg19, and variant calling was performed using a custom-developed analysis tool, as previously described (Iascone *et al.* 2012; Pezzani *et al.,* 2018). Briefly, single nucleotide variants and indels were annotated querying the Genome Aggregation Database, ClinVar (https://www.ncbi.nlm.nih.gov/clinvar/) and Human Gene Mutation Database Professional (HGMD, Release 2020.2). Variants were filtered on the basis of the following criteria: predicted effect on protein and transcript; inheritance model; minor allele frequency in general population (gnomAD MAF threshold ≤ 0.1% or unknown frequency) or in-house database (~4,000 exomes, MAF threshold <1%). For sequencing statistics and data output see **Supplementary Table 6 and 7**. Validation and segregation analysis of the retained variants was carried out by Sanger sequencing.

Supplementary Tables

Supplementary Table 1. Clinical features of the five subjects with pathogenic *ARF3* missense mutations

General information	S1	S2	S3	S4	S5
**Gene**	*ARF3*	*ARF3*	*ARF3*	*ARF3*	*ARF3*
**cDNA change (NM_001659.2)**	c.379A>G	c.34C>G and c.200A>T	c.139C>T	c.277G>A	c.95C>A
**Amino acid change**	p.Lys127Glu	p.Leu12Val and p.Asp67Val	p.Pro47Ser	p.Asp93Asn	p.Thr32Asn
**Mode of inheritance**	sporadic, *de novo*	sporadic, *de novo*	sporadic, *de novo*	sporadic, *de novo*	sporadic, *de novo*
**Gender**	F	M	F	F	M
**Age at last examination**	3 years	18 months	3 years, 10 months	7 years	13 years, 5 months
**Nationality**	Italian	French	Italian	Italian	Italian
**Birth**					
**Gestational age (weeks)**	39	38	41	39	40
**Birth weight (grams/SD)**	2700 g (-1.42 SD)	2800 g (-1.24 SD)	3240 g (-0.73 SD)	3600 g (+0.73 SD)	3500 g (-0.21 SD)
**Birth length (cm/SD)**	46 cm (-2.05 SD)	46.5 cm (-1.73 SD)	not available	50.5 cm (+0.42 SD)	not available
**Head circumference at birth (cm/SD)**	33.5 cm (-1.11 SD)	31.5 cm (-2.33 SD)	not available	33 cm (-0.99 SD)	not available
**Growth**	5 m	16 m	3 y, 10 m	7 y	13 y, 5 m
**Height at last exam (cm/SD, age)**	52 cm (-6.6 SD)	59 cm (-8.21 SD)	101 cm (-0.02 SD)	126 cm (+0.80 SD)	169.2 cm (+1.24 SD)
**Weight at last exam (kg/SD, age)**	3.77 kg (-5.95 SD)	9.7 kg (-1.42 SD)	not available	23.5 Kg (+0.20 SD)	58.1 Kg (+0.94 SD)
**Head circumference at last exam (cm/SD, age)**	37 cm (-4.56 SD)	40 cm (-5.12 SD)	49.4 cm (-1.01 SD)	47.5 cm (-3 SD)	56 cm (+1.5 SD)
**Neurological abnormalities**					
**Developmental delay/Intellectual disability**	yes /profound	yes /profound	yes	yes / severe	yes
**Walking age**	not acquired	not acquired	3 y 9 m	24 m	34 m

**Age at first words**	not acquired	not acquired	not acquired	7 y (two words-sentence)	36 m
**Speech/language development**	absent	absent	delayed	delayed	severe delayed
**Seizures**	yes	no	yes, seizure-free since age 2 y	no	no
**Age at first seizure**	5 m	not applicable	8 m	not applicable	not applicable
**EEG anomalies**	yes, diffuse multifocal epileptic discharges	no	no	not performed	not applicable
**Antiseizure medication**	gardenal (5 mg/Kg/die), baclofen (0.6 mg/Kg/die), lorazepam (2 mg/Kg/die), morphine and clonidine	not applicable	acute intrarectal diazepam (5 mg)	not applicable	not applicable
**Muscle tone**	hypotonia	severe central hypotonia, dystonia	no	hypotonia	no
**Brain MRI (age)**	yes (multiple, 1 m to 3 y)	yes (multiple, 3 m and 14 m)	yes (17 m)	yes (1 y)	yes (3 y 9 m)
**Brain MRI abnormalities**	severe generalized cortical atrophy and ventricular dilatation, severe corpus callosum and brainstem hypoplasia particularly affecting the pons, cerebellar inferior vermis hypoplasia	progressive severe generalized cortical atrophy and ventricular dilatation, severe corpus callosum and brainstem hypoplasia particularly affecting the pons	thin corpus callosum, severe hypomyelination, moderate ventricular dilatation and severe hypoplasia of the anterior part of the temporal lobes	thin corpus callosum	thin corpus callosum, large cisterna magna and severe hypomyelination

**Behavioral anomalies**	not applicable	not applicable	no	no	no
**Additional neurologic findings**	pyramidal signs	pyramidal signs	none	none	none
**Other abnormalities**					
**Eye anomalies**	no	no	no	no	no
**Facial dysmorphism**	bitemporal narrowing, hypotelorism, long eyelashes and flat nasal bridge with small nose	no	no	brachycephaly, deep-set eyes, epicanthal folds, short philtrum, anteverted ears	no
**Palate anomalies (e.g. High p., cleft)**	no	no	no	no	no
**Cardiac anomalies**	pulmonary anomalous venous return, interatrial defect	none	none (echocardiography at 3 y 10 m)	echocardiography not performed	no
**Gastrointestinal problems**	gastrostomy tube	gastrostomy tube	none	none	none
**Urogenital/kidney anomalies**	unilateral pyelectasy, moderate	none	none	none	none
**Skeletal anomalies**	11 pairs of ribs, severe scoliosis	11 pairs of ribs, severe scoliosis	none	X-ray not performed	none
**Other findings**	inguinal hernia	none	neonatal jaundice	pectus excavatum, cafe-au-lait spot (1, leg)	thoracic gibbus

Supplementary Table 2. List of the *ARF3* missense mutations identified in the study

Exon	Nucleotide Change	Amino Acid Change	Subjects	Origin	Metadome d_N/d_S[a]	GERP[b]	CADD phred[c]	MetaSVM[c]	REVEL[c]
2	c.34C>G[d]	p.Leu12Val	2	*de novo*	0.28	4.96	22.8	-0.2469[e]	0.374[e]
2	c.95C>A	p.Thr32Asn	5	*de novo*	0.12	4.96	25.9	1.0941	0.814
2	c.139C>T	p.Pro47Ser	3	*de novo*	0.07	4.96	29.8	0.8949	0.859
3	c.200A>T[d]	p.Asp67Val	2	*de novo*	0.09	4.72	27.3	1.0349	0.918
4	c.277G>A	p.Asp93Asn	4	*de novo*	0.21	5.24	31	0.8948	0.809
4	c.379A>G	p.Lys127Glu	1	*de novo*	0.26	5.35	31	1.0459	0.924

[a] Metadome (stuart.radboudumc.nl/metadome) v1.0.1 tolerance score (d_N/d_S), based on observed (*obs*) missense and synonymous variation in gnomAD.

dN/dS <0.53 indicates intolerance to nonsynonymous variation, <0.18 mean high intolerance at that protein domain position.

[b] Genomic Evolutionary Rate Profiling (GERP) as a measure of sequence conservation across multiple species expressed in terms of 'rejected substitutions'. Positive scores represent a substitution deficit, that is, fewer substitutions are observed than what is expected based on the neutral rate of evolution.

[c] Combined Annotation Dependent Depletion (CADD) v.1.4, MetaSVM and Rare Exome Variant Ensemble Learner (REVEL) scores (sbNSFP v4.1);

values >0 (MetaSVM), >15 (CADD phred) and >0.5 (REVEL) indicate greater likelihood of pathogenicity.

[d]These two variants were in cis.

[e]This score is below the threshold of pathogenicity.

Supplementary Table 3. WES statistics and data output (subject 1)

WES enrichment kit	SureSelect All Exon V7 (Agilent)
Sequencing platform	NovaSeq 6000 (Illumina)
Target regions coverage >4x	96.9%
Target regions coverage >10x	96.4%
Target regions coverage >20x	95.5%
Average depth on target	115x
Total number of high-quality variants	116,088
Variants with effect on CDS or affecting splice sites[1]	16,561
Private, clinically associated and low frequency variants[2]	295
Putative disease genes (autosomal recessive trait)[3]<br>- Filtered candidate genes	2[4]<br>0
Putative disease genes (autosomal dominant trait)[3]<br>- Filtered candidate genes	2[5]<br>*ARF3*

[1]High-quality non-synonymous SNV plus indels within coding exons and splice regions (-/+8).

[2]High-quality, functionally relevant variants and either unknown, private or low frequency variants (gnomAD MAF<0.1% and in house database (approx. population-matched 2,800 exomes) frequency<1%).

[3]Functional impact of variants was assessed by Combined Annotation Dependent Depletion (CADD) v.1.6 (http://cadd.gs.washington.edu/), dbNSFP Mendelian Clinically Applicable Pathogenicity (M-CAP) v.1.0 (http://sites.google.com/site/jpopgen/dbNSFP) and Intervar (http://wintervar.wglab.org) v2.0.1. Variants predicted as benign or likely benign by Intervar were discarded, and only those with CADD score > 15 or M-CAP score > 0.025 were retained.

[4] *SH3TC1* (c.1205G>A, p.Arg402Gln, M-CAP_score: 0.028; c.2267C>T, p.Pro756Leu, M-CAP_score: 0.31), *WDR81* (c.1157T>C, p.Val386Ala, M-CAP_score: 0.025, CADD_score: 27.3; c.3022G>A, p.Val1008Ile, CADD_score: 16.92).

[5] *CACNA1B* (c.390+1_390+2insACGACACGGAGCCCTATTTCATCGGGATCTTTTGCTTCGAGGCAGGGA TCAAAATCATCGCTCTGGGCTTTGTC), *ARF3* (c.379A>G, p.Lys127Glu).

Supplementary Table 4. WES statistics and data output (subject 2)

WES enrichment kit	SureSelect XT Clinical Reasearch Exome, (Agilent)
Sequencing platform	HiSeq4000 (Illumina)
Target regions coverage >4x	98%
Target regions coverage >10x	96%
Target regions coverage >25x	94%
Average depth on target	86x
Total number of high-quality variants	37.761
Variants with effect on CDS or affecting splice sites[1]	9,695
Private, clinically associated and low frequency variants[2]	194
Putative disease genes (autosomal recessive trait)[3]	1[4]
Putative disease genes (de novo trait)[3]<br>- Filtered candidate genes	1[5]<br>*ARF3*

[1]High-quality non-synonymous SNVs plus indels within coding exons and splice regions (-/+14).

[2]High-quality, functionally relevant variants and either unknown, private or low frequency variants (gnomAD MAF<0.1% and frequency<1% within Integragen database).

[3]Functional impact of variants was assessed by Combined Annotation Dependent Depletion (CADD) v.1.4 (http://cadd.gs.washington.edu/), dbNSFP Mendelian Clinically Applicable Pathogenicity (M-CAP) v.1.0 (http://sites.google.com/site/jpopgen/dbNSFP) and Intervar (http://wintervar.wglab.org) v2.0.1. Variants predicted as benign or likely benign by Intervar were discarded and only those with CADD score>15 or M-CAP score>0.025 were retained.

[4] *TMEM131* (c.1208A>G, p.Gln403Arg, CADD score: 17.130, M_CAD score: 0.012; c.878C>T; p.Ala293Val, CADD score: 19.640, M_CAD score: 0.027)

[5] *ARF3* (c.34C>G, p.Leu12Val, CADD: score 11, M_CAD score: 0.075; c.200A>T, p.Asp67Val, CADD score: 17.190, M_CAD score: 0.806; in *cis*).

Supplementary Table 5. WES statistics and data output (subject 3)

WES enrichment kit	Human Core Exome Kit + RefSeq V1 (Twist Bioscience)
Sequencing platform	NextSeq 550 (Illumina)
Target regions coverage >5x	97.7%
Target regions coverage >10x	97.6%
Target regions coverage >20x	97.5%
Average depth on target	97.3x
Total number of high-quality variants	127,887
Variants with effect on CDS or affecting splice sites[1]	22,153
Private, clinically associated and low frequency variants[2]	748
Putative disease genes (autosomal recessive trait)[3]	0
Putative disease genes (de novo trait)[3]<br>- Filtered candidate genes	3[4]<br>*ARF3**

[1]High-quality non-synonymous SNVs plus indels within coding exons and splice regions (-/+8).

[2]High-quality, functionally relevant protein-altering variants and either unknown, private or low frequency variants (gnomAD MAF<0.1%).

[3]frequency <1% within our ~internal database, non-homozygous in allele frequency databases (gnomAD)

[4]heterozygous variants in disease-genes inherited from a healthy parent: *KCNH1*, NM_172362.2:c.2359G>A, maternal; *CACNA1A*, NM_001127221.1:c.6104G>A, paternal.

* *de novo ARF3* (NM_001659.2:c.139C>T, p.Pro47Ser).

Supplementary Table 6. WES statistics and data output (subject 4)

WES enrichment kit	SureSelect Clinical Research Exome V2 (Agilent)
Sequencing platform	NextSeq 500 (Illumina)
Target regions coverage >4x	99.5%
Target regions coverage >10x	98.4%
Target regions coverage >25x	96.4%
Average depth on target	252x
Total number of high-quality variants	116,152
Variants with effect on CDS or affecting splice sites[1]	15,490
Private, clinically associated and low frequency variants[2]	1,863
Putative disease genes (autosomal recessive trait)[3]	NA
Putative disease genes (de novo trait)[3]<br>- Filtered candidate genes	1[3]<br>*ARF3*

[1]High-quality non-synonymous SNVs plus indels within coding exons and splice regions (-/+8).

[2]High-quality, functionally relevant variants and either unknown, private or low frequency variants (gnomAD MAF<0.1% and frequency<1% within our exomes database).

[3]*ARF3*: Chr12(GRCh37):g.49333545C>T, NM_001659.2:c.277G>A; p.(Asp93Asn), *de novo*; CADD score: 31.

Supplementary Table 7. WES statistics and data output (subject 5)

WES enrichment kit	SureSelect Clinical Research Exome V2 (Agilent)
Sequencing platform	NextSeq 500 (Illumina)
Target regions coverage >4x	99.7%
Target regions coverage >10x	98.1%
Target regions coverage >25x	95.3%
Average depth on target	212x
Total number of high-quality variants	114,803
Variants with effect on CDS or affecting splice sites[1]	15,235
Private, clinically associated and low frequency variants[2]	1,752
Putative disease genes (autosomal recessive trait)	NA
Putative disease genes (de novo trait)<br>- Filtered candidate genes	1[3]<br>*ARF3*

[1]High-quality non-synonymous single nucleotide variants plus indels within coding exons and splice regions (-/+8).

[2]High-quality, functionally relevant variants and either unknown, private or low frequency variants (gnomAD MAF<0.1% and frequency<1% within our exomes database).

[3]*ARF3*: Chr12(GRCh37):g.49334784G>T, NM_001659.2:c.95C>A, p.Thr32Asn, de novo; CADD score: 25.6.

Supplementary Table 8. Pathogenic variants in ARF3 paralogs, and HRAS, NRAS and KRAS GTPases affecting codons corresponding and/or adjacent to the residues mutated in ARF3. Equivalent residues, disease-causing amino acid substitutions and associated traits are indicated.

ARF3	ARF1	ARF4	ARF5	ARF6	HRAS	KRAS	NRAS
Thr32<br>**Thr32Asn**	Thr32	Thr32	Thr32	Thr28	Ala18<br>Ala18Thr/Val (somatic)<br>Mutation hotspot involving Gly12-Ser17 (somatic, COSMIC)	Ala18<br>Ala18<br>Mutation hotspot involving Gly10-Val14 (somatic, COSMIC)	Ala18<br>Ala18Thr (somatic, COSMIC)
Pro47<br>**Pro47Ser**	Pro47<br>Thr48Ile<br>(NDD, ClinVar)	Pro47	Pro47<br>Ile46Val (somatic, COSMIC)	Pro43	Pro34<br>Pro34Ser (somatic, COSMIC)	Pro34<br>Pro34Leu/Arg (RASopathy)[5]<br>Pro34Thr/Ser/Arg/Leu (somatic, COSMIC)	Pro34<br>Pro34Leu (NS) (ClinVar)<br>Pro34Leu/Arg (somatic, COSMIC)
Asp67<br>**Asp67Val**	Asp67	Asp67<br>Asp67Asn (somatic)<br>Trp66Leu (somatic) (COSMIC)	Asp67<br>Asp67Tyr (somatic)<br>Mutation hotspot involving Val68-Gly70 (somatic) (COSMIC)	Asp63<br>Trp62Cys (somatic) (COSMIC)	Asp57<br>Thr58Ile (CS)[3]<br>Asp57Asn (somatic)<br>Mutation hotspot involving Thr58-Gln61 (somatic) (COSMIC)	Asp57<br>Thr58Ile (NS)[4]<br>Asp57Asn (somatic)<br>Mutation hotspot involving Thr58-Gln61 (somatic) (COSMIC)	Asp57<br>Asp57Asn/Ala/Glu (somatic)<br>Mutation hotspot involving Thr58-Gln61 (somatic) (COSMIC)
Asp93<br>**Asp93Asn**<br>Asp93Asn (somatic, COSMIC)	Asp93	Asp93<br>Asp93Glu (somatic)<br>Mutation hotspot involving Asn95-Arg99 (somatic) (COSMIC)	Asp93<br>Ser94Gly (somatic)<br>Mutation hotspot involving Asp96-Arg99 (somatic) COSMIC)	Asp89	Ala83<br>Ala83Asp (somatic) (COSMIC)	Ala83<br>Ile84Met (somatic) (COSMIC)	Ala83
Lys127<br>**Lys127Glu**<br>Asn126Ser (somatic, COSMIC)	Lys127<br>Lys127Glu (NDD)[1]	Lys127<br>Asn126Lys (somatic) (COSMIC)	Lys127	Lys123	Lys117<br>Lys117Arg (CS)[2]<br>Lys117Asn/Glu (somatic, COSMIC)	Lys117<br>Lys117Asn/Arg/Tyr/Glu (somatic, COSMIC)	Lys117

NDD, neurodevelopmental disorder; NS, Noonan syndrome; CS, Costello syndrome. COSMIC database (https://cancer.sanger.ac.uk/cosmic/); ClinVar database (https://www.ncbi.nlm.nih.gov/clinvar/).

[1] Ge et al. 2016, NPJ Genom Med, 1:16036 [PMID: 28868155].

[2] Kerr et al. 2006, J Med Genet, 43: 401-5 [PMID: 16443854]; Denayer et al., 2008, Hum Mutat, 29:232-9 [PMID: 17979197].

[3] Gripp et al. 2012, Am J Med Genet Part A, 158A:1095-101 [PMID: 22488832].

[4] Schubbert et al. 2006, Nat Genet, 38:331-6 [PMID: 16474405]; Nava et al. 2007, J Med Genet, 44:763-71 [PMID: 17704260].

[5] Schubbert et al. 2006, Nat Genet, 38:331-6 [PMID: 16474405]; Zenker et al. 2007, J Med Genet, 44:131-5 [PMID: 17056636].

[6] Zenker et al. 2007, J Med Genet, 44:131-5 [PMID: 17056636]; Bertola et al. 2007, J Hum Genet, 52:521-526 [PMID: 17468812]; Nava et al. 2007, J Med Genet, 44:763-71 [PMID: 17704260]; Bertola et al. 2012, Case Reports, 81:595-7 [PMID: 22211815].

Supplementary Table 9. ARF3-GTP hydrogen bonds present for more than 50% of simulation time in the ARF3:COPG1-COPZ1 complex considering ARF3^{WT}, ARF3^{D67V} and ARF3^{P47S}

Donor residue	Donor atom	Acceptor res	Acceptor atom	% res time		
				WT	Asp67Val	Pro47Ser
GTP	N2	ASP129	OD2	73.2	71.9	73.7
ALA27	N	GTP	O1B/O2G/O3B	84.4	92.1	81.9
THR31	N	GTP	O1A/O2B	99.6	98.7	99.8
THR32	OG1	GTP	O5'/O1A	73.6	95.5	98.3
THR45	OG1	GTP	O1A/O1B	99.8	*10.7*	79.1
THR48	N	GTP	O1G	97.6	95.2	83.7
LYS127	NZ	GTP	O4'/N9	73.2	*35.5*	74.8
LYS30	N	GTP	O1B/O2B	*62.4*	99.9	99.8
LYS30	NZ	GTP	O2G/O3B	*5.8*	96.2	98.5

Supplementary Table 10. ARF3:COPG1 hydrogen bonds present for more than 40% of simulation time in the ARF3:COPG1-COPZ1 complex considering ARF3^{WT}, ARF3^{D67V} and ARF3^{P47S}. ARF3 residues are highlighted in bold.

Donor residue	Donor atom	Acceptor res	Acceptor atom	% res time		
				WT	Asp67Val	Pro47Ser
**ARG19**	NH1	PRO38	O	51.8		
**ARG19**	NH2	ASN35	O	70.9		
**THR48**	OG1	SER107	OG	69.3		
**ASN84**	ND2	THR37	N	69.5		
**ARG19**	NH1	THR37	O		45.2	
ARG32	NH2	**HIS80**	ND1		93.0	
**LYS38**	NZ	ASN80	OD1			43.5
**THR44**	N	TYR143	OH			48.6
SER107	N	**PHE51**	O			40.5

Supplementary Table 11. List of primers used in this stu

Gene	Primer sequence (5' – 3')	Application, size (bps)
arf3a	FW: TATCCGGTGTCTGTGCCTGAGG	RT-PCR (1-2 hpf), 445
	RW: GCAGCATTCATGGCATTTGG	
arf3b	FW: GACTCGCAGCTTTAAGGACG	RT-PCR (1-2 hpf), 627
	RW: TAATCCCACCCGTTCCCAAA	
arf3a	FW: TATCCGGTGTCTGTGCCTGAGG	RT-PCR (2.5 - 18 hpf), 748
	RW: TGGGGTGTAGTGAGGGGATA	
arf3b	FW: GACTCGCAGCTTTAAGGACG	RT-PCR (2.5 - 18 hpf), 627
	RW: TAATCCCACCCGTTCCCAAA	
eif1α	FW: CTTCTCAGGCTGACTGTGC	RT-PCR, 358
	RW: CCGCTAGCATTACCCTCC	
ARF3-myc	FW: GACAAGATTCGACCCCTCTG	RT-PCR, 399
	RW: CGGTATGCATATTCAGATCCTC	
GAPDH	FW: CTACACTGAGCACCAGGTG	RT-PCR, 222
	RW: CCTCTTGTGCTCTTGCTGG	
arf3a_ATG MO	AAGGTTGCCAAAAATGTTCCCCATG	MO injection
arf3b_ATG MO	GCAGATTGCCAAAAATATTCCCCAT	MO injection
krox-20	FW: GTGCACCCTCTTGCCGATAG	*in situ* hybridization
	RW: GGACGCAGGATTGGCCTGAG	
myoD	FW: CCTTGCTTCAACACCAACGA	*in situ* hybridization
	RW: AAATGGTTTCCTGAGCCTGC	
T7	FW: TAATACGACTCACTATAGGG	sequencing
SP6	FW: ATTTAGGTGACACTATAG	sequencing
ARF3_ex2	FW: CCCCACAAATTGTATGGACTT	Sanger sequencing of p.P47S variant
ARF3_ex2	RW: TCTTCCATACCAGGGACCAG	
ARF3_4F	FW: GGTGGCCAACTGTTACCCTG	Sanger sequencing of p.K127E variant
ARF3_4R	RW: AGCCAACAATTTCCACAGCC	

Supplementary Table 12. Overview of the statistical analyses

Figures	Analyses	Statistical details*
2	Expression levels of Myc-tagged ARF3 proteins in COS-1 cells with or without CHX treatment (a)	Two-way ANOVA with Tukey's *post hoc* test, Interaction: F (6,24) = 1.934, Treatment: F (2,24) = 15.39, Genetic condition: F (3,24) = 304.6<br><br>Two-way ANOVA with Tukey's *post hoc* test, Interaction: F (4,18) = 0.1103, Treatment: F (2,18) = 0.5074, Genetic condition: F (2,18) = 1.542
	Expression levels of Myc-tagged ARF3 proteins in COS-1 cells after treatment with MG132 and Bafilomycin (a')	Two-way ANOVA with Tukey's *post hoc* test, Interaction: F (4,18) = 2.028, Treatment: F (2,18) = 9.520, Genetic condition: F (2,18) = 7.502
	Pulled-down assay in COS-1 cells (b)	One-way ANOVA with Dunnett's *post hoc* test, F (7,16) = 58.75
3	Golgi morphology in COS-1 cells (c)	Two-sided Chi-square's test in a 2×2 contingency table
5	COPI assembly in COS-1 cells (b)	Two-sided Chi-square's test in 2x2 contingency table
6	Tfn intracellular distribution in COS-1 cells (b)	Two-sided Chi-square's test in 2x2 contingency table
	Clustered Tfn at PN in COS-1 cells (b')	One-way ANOVA with Dunnett's *post hoc* test,<br>F (5, 7) = 13.01
	Tfn (%) colocalization with Rab 5 in COS-1 cells (d)	One-way ANOVA with Dunnett's *post hoc* test, F (5, 86) = 13.92
7	Survival rate in zebrafish embryos (c')	Log-rank (Mantel-Cox) test
	Gross phenotype in ARF3-overexpressing and MO-injected embryos at 24 and 48 hpf (c''-e'')	Two-sided Chi-square's test in 2x2 contingency table
8	Head area measurements at 48 hpf (b, left panel)	One-way ANOVA with Dunnett's *post hoc* test, F (3, 91) = 14.72
	Head area measurements at 48 hpf (b, right panel) and 4.5 dpf (Fig. 8b')	Non-parametric Kruskal-Wallis with Dunn's post hoc test
	Anterior brain volume at 48 hpf (c', left panel)	Unpaired t-test with Welch's *post hoc* test, t=4.586, df=3.252
	Anterior brain volume at 48 hpf (c', right panel)	One-way ANOVA with Dunnett's *post hoc* test, F (3, 10) = 10.78
9	Number of $pH3^+$ cells (c, upper panel)	One-way ANOVA with Dunnett's *post hoc* test, F (2, 12) = 4.581
	Number of $pH3^+$ (c, lower panel)	One-way ANOVA with Dunnett's *post hoc* test, F (3, 16) = 1.146
	Incidence of $pH3^+$ cells at different mitotic stages (e)	Two-sided Chi-square's test in 2x2 contingency table
	Number of AO^+ spots at 48 hpf (g, upper panel)	One-way ANOVA with by Dunnett's *post hoc* test, F (5, 29) = 2.903

	Number of AO^+ spots at 48 hpf (g, lower panel)	Kruskal-Wallis with by Dunn's *post hoc* test
10	Notochord curvatures at 30 hpf (c, right panel)	Two-sided Chi-square's test in 2x2 contingency table
	Number of notochord curvatures at 30 hpf (c', left panel)	Non-parametric Kruskal-Wallis with Dunn's *post hoc test*
	AP embryo extension at 15 hpf (f)	Non-parametric Kruskal-Wallis with Dunn's *post hoc test*
	CE index at 15 hpf (g)	Two-sided Chi-square's test in 2x2 contingency table
Suppl.4	ARF3 protein levels in zebrafish embryos (b')	Two-way ANOVA with Tukey's post hoc test, Time: F (1, 2) = 3.794, Genetic conditions: F (2, 2) = 224.9
Suppl.5	Total fluorescent intensity of ARF3mCherry-positive COS-1 cells (b')	Non-parametric Mann-Whitney with Dunn's *post hoc* test
	Total fluorescent intensity of ARF3mCherry-positive COS-1 cells (c')	Non-parametric Kruskal-Wallis with Dunn's *post hoc* test
Suppl. 10	Golgi morphology in zebrafish embryos (a, c)	Two-sided Chi-square's test in 2x2 contingency table
Suppl. 11	Tfn distribution in COS-1 cells upon 5' of treatment (b)	Two-sided Chi-square's test in 2x2 contingency table
	Clustered Tfn distribution in COS-1 cells upon 5' of treatment (b')	One-way ANOVA with Dunnett's *post hoc* test, F (5, 6) = 5.000
	Tfn distribution in NT COS-1 cells upon 30' of treatment (c)	Two-sided Chi-square's test in 2x2 contingency table
Suppl. 12	Tnf (%) colocalization with Rab 11 in COS-1 cells (b)	Kruskal-Wallis with Dunn's *post hoc* test
Suppl. 13	Tfn (%) colocalization with Lamp2 in COS-1 cells (b)	Kruskal-Wallis with Dunn's *post hoc* test Unpaired t-test, t=0.02832, df=17 (WT and T32N, right panel)
	Lamp2 (%) colocalization with Tfn in COS-1 cells (c)	One-way ANOVA with Dunnett's *post hoc* test, F (4, 42) = 8.133 Unpaired t-test, t=3.334, df=17 (WT and T32N, right panel)
Suppl. 15	Gross phenotype at 48 hpf (a)	Two-sided Chi-square's test in 2x2 contingency table
	Survival rate in MO-injected zebrafish embryos (b)	Log-rank (Mantel-Cox) test
Suppl. 16	Anterior brain volume at 48 hpf (c)	Two-tailed unpaired Welch's t-test, t=4.661, df=7.515
Suppl. 17	Width of lateral Fb bundles at 48 hpf (b, left panel)	One-way ANOVA with by Dunnett's *post hoc* test, F (3, 19) = 14.50
	Width of lateral Fb at 48 hpf (b, right panel)	One-way ANOVA with by Dunnett's *post hoc* test, F (3, 19) = 14.50
	Width of medial AC at 48 hpf (c, left panel)	One-way ANOVA with by Dunnett's *post hoc* test, F (3, 19) = 4.566
	Width of medial AC at 48 hpf (c, right panel)	Non-parametric Kruskal-Wallis with by Dunn's *post hoc* test

	Distribution of pH3$^+$ within the OT at 48 hpf (h)	Two-sided Chi-square's test in 2x2 contingency table
Suppl. 18	Ectopic pH3$^+$ within the dorsal anterior brain at 48 hpf (j, left panel)	One-way ANOVA with Dunnett's *post hoc* test, F (2, 12) = 0.9090
	Ectopic pH3$^+$ within the dorsal anterior brain at 48 hpf (j, right panel)	One-way ANOVA with Dunnett's *post hoc* test, F (3, 16) = 4.360
	Ectopic pH3$^+$ within the left OT at 48 hpf (j, left panel)	One-way ANOVA with Dunnett's *post hoc* test, F (2, 12) = 0.09258
	Ectopic pH3$^+$ within the left OT at 48 hpf (j, right panel)	One-way ANOVA with Dunnett's *post hoc* test, F (3, 16) = 2.525
	Ectopic pH3$^+$ within the right OT at 48 hpf (j', left panel)	One-way ANOVA with Dunnett's *post hoc* test, F (2, 12) = 0.2607
	Ectopic pH3$^+$ within the right OT at 48 hpf (j', right panel)	One-way ANOVA with Dunnett's *post hoc* test, F (3, 16) = 0.6828
	Ectopic pH3$^+$ within the at 48 hpf (k, left panel)	Non-parametric Kruskal-Wallis with Dunn's *post hoc* test
	Ectopic pH3$^+$ within the Ce at 48 hpf (k, right panel)	One-way ANOVA with Dunnett's *post hoc* test, F (3, 16) = 8.243
Suppl. 19	Mitotic spindle width at 28 hpf (d, left panel)	Two-tailed unpaired t-test, t=2.629, df=44,
	Mitotic spindle width at 28 hpf (d, right panel)	Two-tailed unpaired t-test, t=1.528, df=30
	Mitotic spindle lenght at 28 hpf (d', left and right panel)	Non-parametric Mann-Whitney test
Suppl. 20	Number of somites at 15 hpf	Mixed-effects Two-way ANOVA with Sidak's *post hoc* test, Left and right: F (1, 246) = 0.01952, Genetic conditions F (6, 246) = 14.41, Interaction: F (6, 246) = 1.197
Suppl. 21	Angle between A-P ends at 13 hpf (c)	Non-parametric Kruskal-Wallis with Dunn's *post hoc* test
	Number of cell protrusions (f)	Mixed-effects Two-way ANOVA with Sidak's *post hoc* test, Time: F (2.352, 42.34) = 4.417, Genetic conditions F (1, 18) = 7.149, Interaction: F (3, 54) = 8.546

* "n" values are provided in the relative figure legends

Supplementary Figures

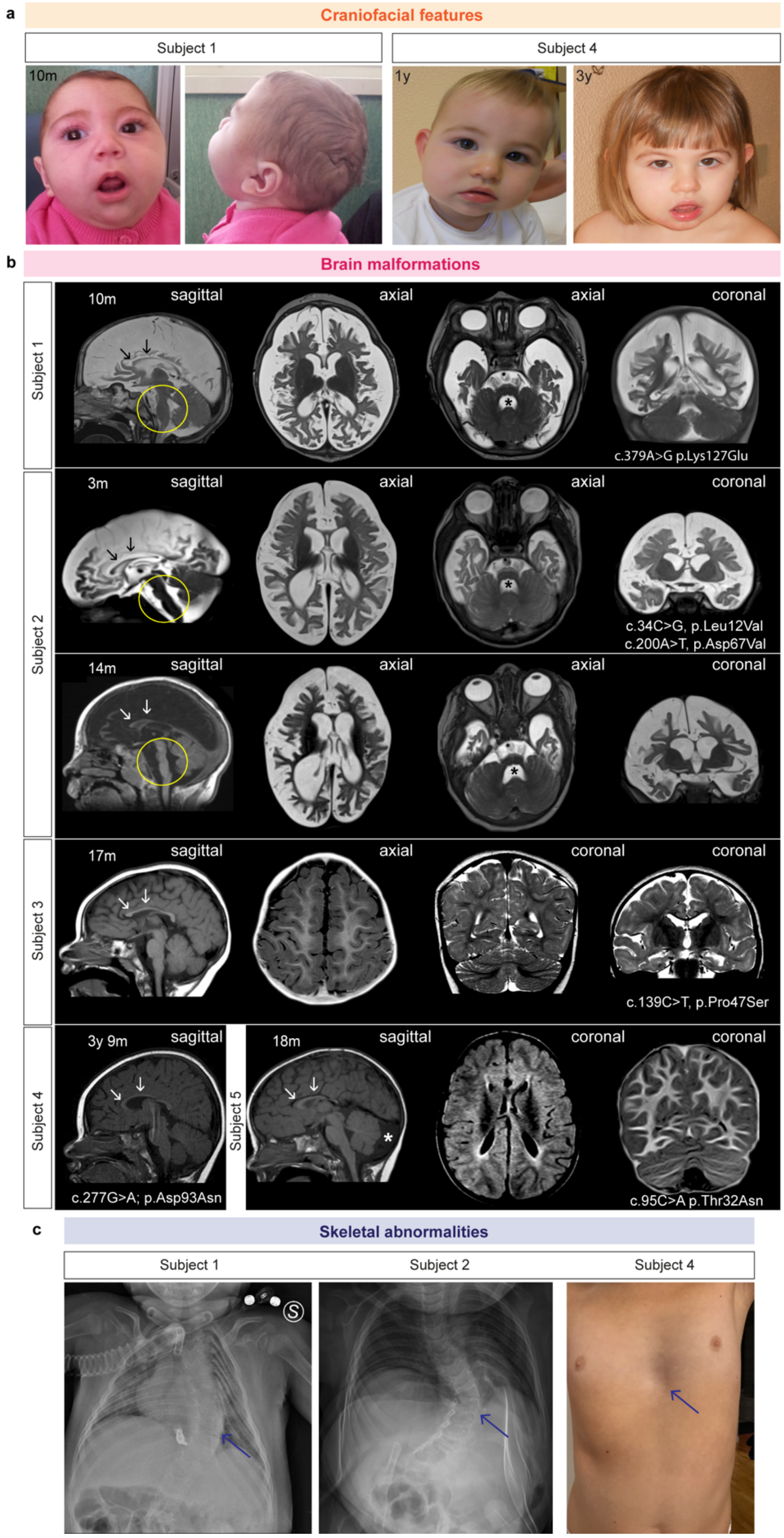

Supplementary Figure 1. Clinical features of individuals with *de novo ARF3* mutations. (**a**) facial features of Subjects 1 and 4. The craniofacial appearance of Subject 1 is characterized by microcephaly, bitemporal narrowing, hypotelorism, long eyelash es, cheeks hypotonia, and flat nasal bridge with small nose. Subject 4 shows deep-set eyes, epicanthal folds, cheeks hypotonia, short nose with anteverted nares, deep philtrum and everted lower lip. (**b**) brain MRI of all the subjects described in this study at different ages. All images for S1 and S2 in row 1 to 3 are T2 weighed (T2W), except the first image on the left of S2 at 14 months, which is T1W (3rd row). In these two patients, the first images on the left are sagittal, those in the center are axial and those in the right end are coronal. S1 and S2 show very similar findings with severe generalized cortical atrophy and ventricular dilatation, severe corpus callosum (arrows in the sagittal image) and brainstem hypoplasia (encircled in yellow) particularly affecting the pons. The cerebellar hemispheres are spared by the atrophy, the inferior vermis however is partially involved, and the resulting surface of the 4th ventricle is considerably enlarged (black asterisk in the axial images). An obvious progression of the atrophy can be observed in S2 between 3 and 14 months. The 4th horizontal row shows MRI scans from S3 at 17 months. The 1st image on the left is sagittal and T1W, the 2nd image is axial and T1W, the 3th image is axial and T2W, while the 4th image is coronal and T2W. S3 shows a thin corpus callosum (arrows in the first sagittal image), severe hypomyelination (well visible as high signal intensity of the white matter in the 3rd and 4th mages), moderate ventricular dilatation (not shown), particularly involving the temporal horns (4th image on the right) and severe hypoplasia of the anterior part of the temporal lobes, (3rd and 4th images). Note the retracted temporal lobes with overlying large subarachnoid space (stars in the 3rd and 4th scans). In the last row T1W images are shown of S4 at 3 years and 9 months (sagittal cut on the left), showing a thin corpus callosum and S5 at 18 months (the other three images, the 2nd is an axial FLAIR cut and the 3rd is a T1W coronal cut). S5 exhibits a thin corpus callosum (arrows) and a large cisterna magna (white asterisk) and severe hypomyelination (high signal intensity of the white matter in the 2nd image, and as low signal intensity in the 3rd image). (**c**) documentation of the skeletal malformations of S1, S2, S4. Chest X-Ray of: S1 and S2 show severe scoliosis and 11 pairs of ribs. A clinically significant pectus excavatum is observed in S4.

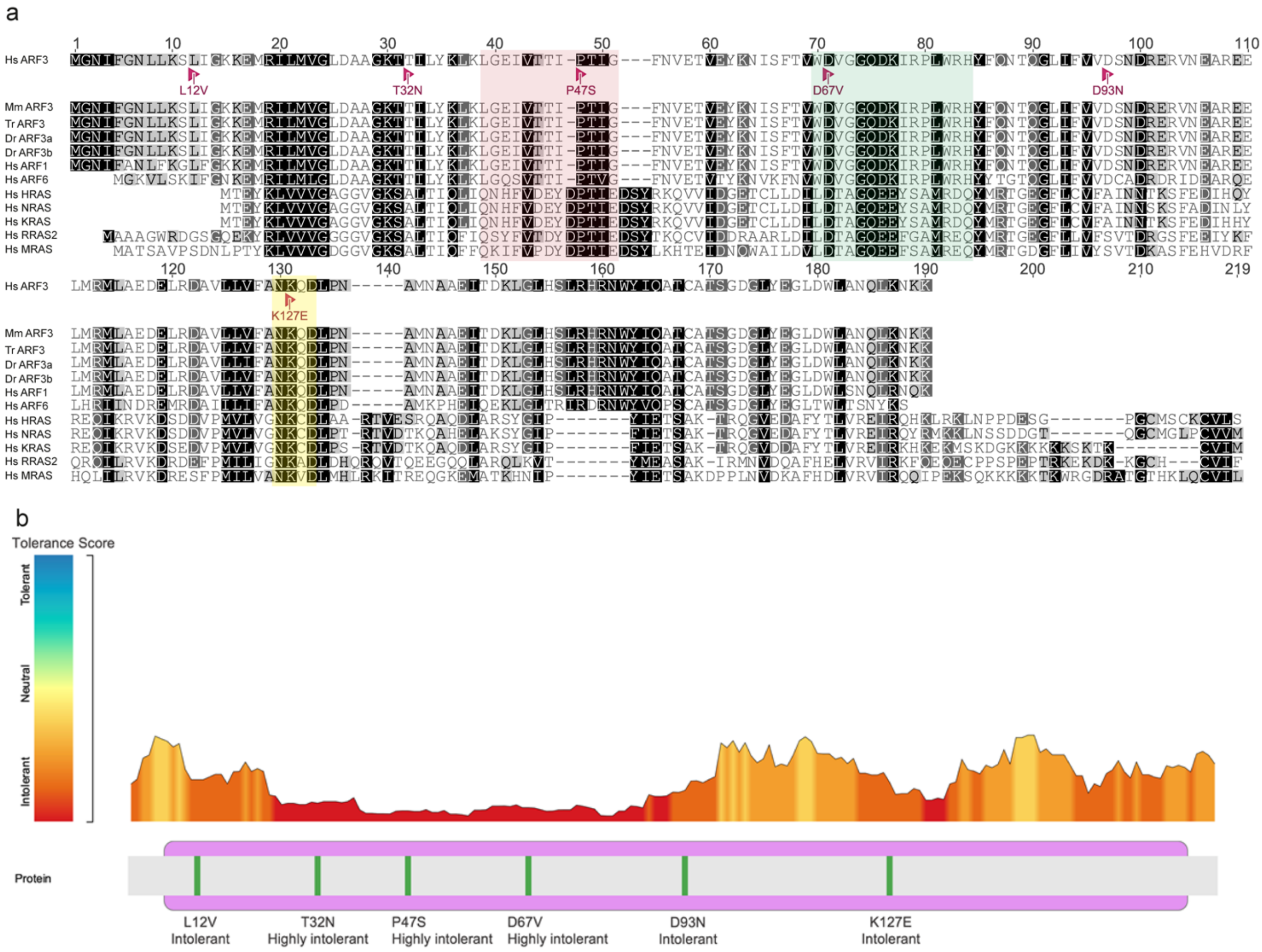

Supplementary Figure 2. ARF3 multiple sequence alignment and mutations tolerance landscape. (**a**) Multiple alignment of the amino acid sequences of ARF3, ARF1, ARF6, HRAS, NRAS, RRAS2, MRAS from human (Hs: *Homo sapiens*) compared to ARF3 sequences from *Mus musculus (Mm)*, *Takifugu rubripes* (Tr) and *Danio rerio* (Dr). The alignment was obtained with MUSCLE v.5 (EBI). The patient mutations are indicated (purple triangles) in the comparison and show conservation across ARF sequences. The mutations p.P47S, p.D67V and p.K127E are located in crucial functional domains of the proteins (red: switch1, green: switch2, yellow: NKXD motif, refer to main Fig.1) and are shared between ARF and RAS families. Sequence data for multiple protein alignment were retrieved from NCBI: human NRas: NP_002515.1 (https://www.ncbi.nlm.nih.gov/search/all/?term=NP_002515.1); human M-RAS isoform 1 precursor: NP_001078518.1 (https://www.ncbi.nlm.nih.gov/search/all/?term=NP_001078518.1); human K-RAS isoform b : NP_004976.2 (https://www.ncbi.nlm.nih.gov/search/all/?term=NP_004976.2); zebrafish arf3b : NP_001012248.1 (https://www.ncbi.nlm.nih.gov/search/all/?term=NP_001012248.1); zebrafish arf3a : NP_001003441.1

(https://www.ncbi.nlm.nih.gov/search/all/?term=NP_001003441.1); human RRAS2 isoform a: NP_036382.2 (https://www.ncbi.nlm.nih.gov/search/all/?term=_NP_036382.2) and from Uniprot: mouse ARF3: P61205 (https://www.ncbi.nlm.nih.gov/search/all/?term=_NP_036382.2); human HRAS : P01112 (https://www.uniprot.org/uniprotkb/P01112/entry); human ARF6: P62330 (https://www.uniprot.org/uniprotkb/P62330/entry); human ARF3 : P61204 (https://www.uniprot.org/uniprotkb/P61204/entry); human ARF1: P84077(https://www.uniprot.org/uniprotkb/P84077/entry). (**b**) Tolerance landscape for the ARF3 meta-domain. The analysis, conducted on the aggregation of variation data across homologous domains, highlights a low tolerance profile to missense mutations along the whole ARF3 protein domain (PF00025, violet). As shown, the five altered residues (marked green) occupy intolerant/high intolerant positions, with considerably low nonsynonymous$_{obs}$/synonymous$_{obs}$ ratios.

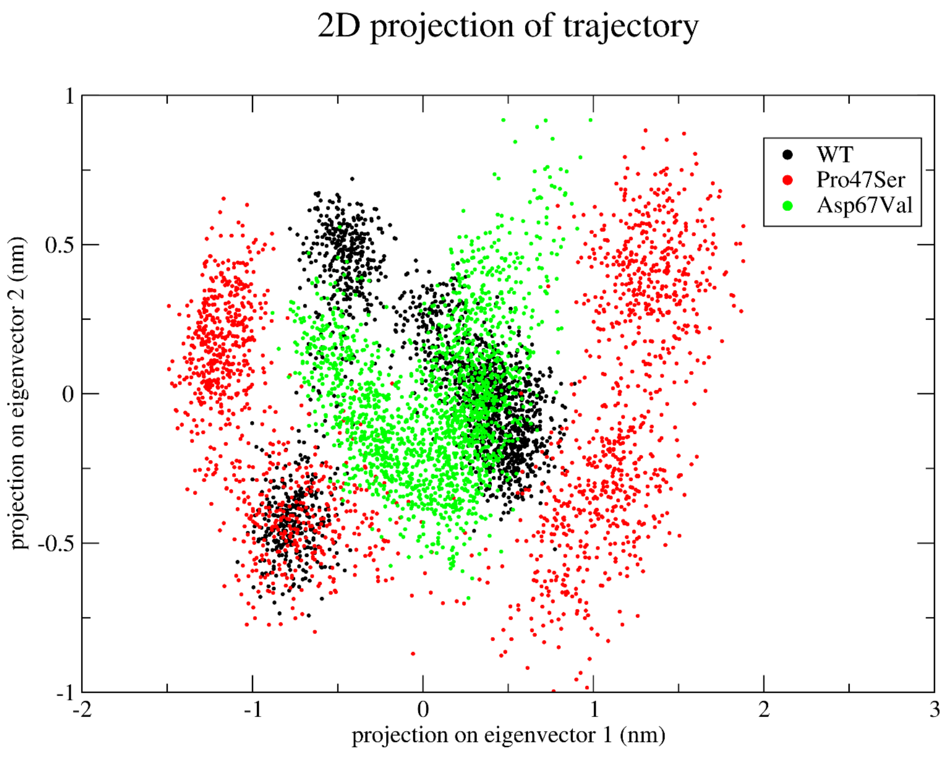

Supplementary Figure 3. 2D projection of ARF3^{WT}, ARF3^{D67V} and ARF3^{P47S} on the respective essential subspace along eigenvectors 1 and 2 resulting from MD simulations. The Essential Dynamics analysis, technique is based on the diagonalization of the covariance matrix built from the atomic fluctuations after the removal of the translational and rotational movement, and it is very effective in separating the large collective protein global motions from the small uninteresting fluctuations (Amadei *et*

al., 1993). The Pro47Ser system shows an enlarged conformational basin visited in the 500 ns MD simulation as compared to WT and Asp67Val. Comparison of Figure 1h (Pro47Ser RMSF) vs 1f-g (WT and Asp47Val RMSF, respectively) shows that the region most perturbed in Pro47Ser involve the ARF3-COPG1 interface.

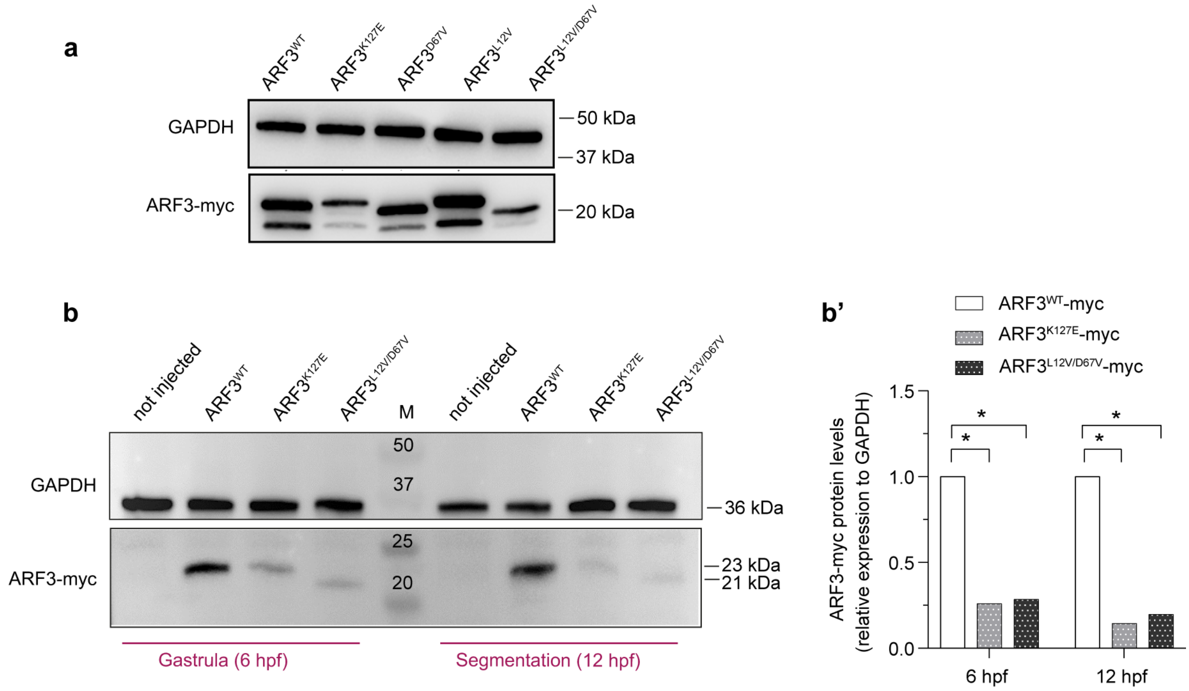

Supplementary Figure 4. Myc-tagged ARF3 protein levels in COS-1 cells and zebrafish embryos during gastrulation and segmentation stages. (**a**) Western blot analysis showing the level of expression of ARF3 protein from COS-1 cells transfected with WT or mutant myc-tagged ARF3 DNA constructs (K127E, L12V/D67V). Expression of ARF3 carrying the single substitutions found in cis (L12V and D67V) are also tested. ARF3^{D67V} (3rd lane) migrates faster compared to ARF3^{WT}, ARF3^{L12V} and ARF3^{K127E}, likely due to the "gel shifting" phenomenon (Shi et al., 2012) caused by an increased binding to SDS molecules and a change of the electrophoretic mobility. Blots are representative of four independent experiments for WT, K127E and L12V/D67V and a single experiment for L12V and D67V. (**b-b'**) Western blot analysis showing the levels of expression of ARF3 in zebrafish from total extracts of not injected fish and fish injected with mRNA encoding myc-tagged ARF3^{WT} or mutant myc-tagged ARF3 (K127E, L12V/D67V). In fish expressing mutant ARF3 the protein levels are reduced starting already from gastrula stage, sustained degradation is observed at later time points (6 and 12 hpf, ARF3^{K127E} *p=0.0107, ARF3$^{L12V/D67V}$ *p=0.016). in b protein extracts from the same experiment are loaded on different blots and processed parallelly for GAPDH (reference gene, ~36 kDa), and myc-tagged ARF3 (~23 kDa). Blot (b) and quantification (b') of ARF3 protein levels derive from embryo protein extracts of one batch. Data are expressed as

relative protein expression (fold change) to GAPDH control protein and Two-way ANOVA followed by Tukey's multiple comparisons post hoc test is used. Source data are provided as a Source Data file.

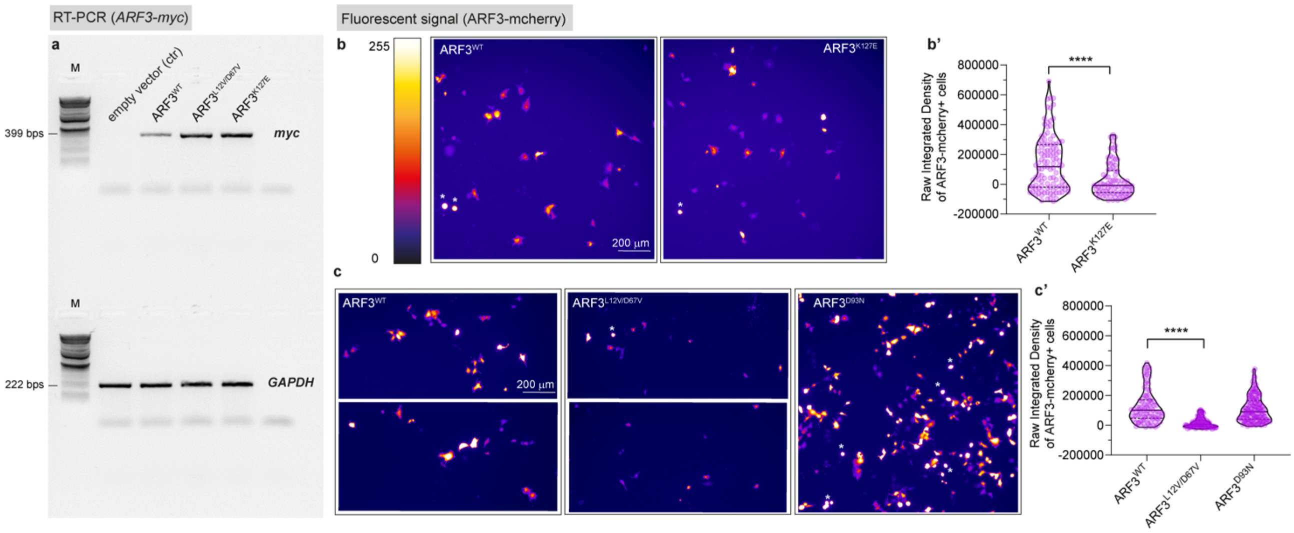

Supplementary Figure 5. RT-PCR of myc-tagged ARF3^{WT}, ARF3 $^{L12V/D67V}$ and ARF3^{K127E} and quantification of total fluorescent intensity of mCherry tagged-ARF3^{K127E}, ARF3$^{L12V/D67V}$ and ARF3^{D93N} upon overexpression in COS-1 cells. (**a**) RT-PCR from total RNA of COS-1 cells transfected with the empty vector or myc-tagged ARF3 ^{WT} and mutants (K127E and L12V/D67V) for 48h showing no significant change in the relative abundance of transcribed mRNA of ARF3^{K127E} and ARF3$^{L12V/D67V}$ mutants compared to ARF3^{WT}. GAPDH is used as housekeeping gene. The raw uncropped agarose gel shows the result of a single RT-PCR experiment. (**b**) Microscopy images of COS-1 cells expressing mCherry-tagged ARF3^{WT} and ARF3^{K127E}. (**b'**) Quantification of total fluorescent intensity (measured as raw integrated density) of mCherry-tagged ARF3. N of cells: 109 (WT), 82 (K127E, **** $p<0.0001$). A statistically significant decrease in mCherry signal is observed for K127E compared to WT. (**c**) Microscopy images of COS1 cells expressing mCherry-tagged ARF3^{WT} and mutants (K127E and L12V/D67V). For WT and L12V/D67V two representative fields are shown. Images in b and c are representative of at least three independent experiments and are shown in a "Fire" LUT mode (obtained by Fiji) (**c'**) Quantification of total fluorescent intensity (measured as raw integrated density) of mCherry-tagged ARF3. N of cells: 88 (WT), 100 (L12V/D67V, **** $p<0.0001$), 149 (D93) of a single experiment. A statistically significant decrease signal is observed in L12V/D67V compared to WT. Round-shaped bright positive cells considered as possible apoptotic cells are showed with white asterisks (b) and were excluded for the

fluorescence analysis. In b’ and c’ data are expressed as violin plot with median (middle line), 25th–75th percentiles (dotted lines). All data points are shown. Non-parametric Mann-Whitney's test (b’) and Kruskal-Wallis followed by Dunn’s multiple comparison *post hoc* test are used to assess the statistical significance (c’). Source data are provided as a Source Data file.

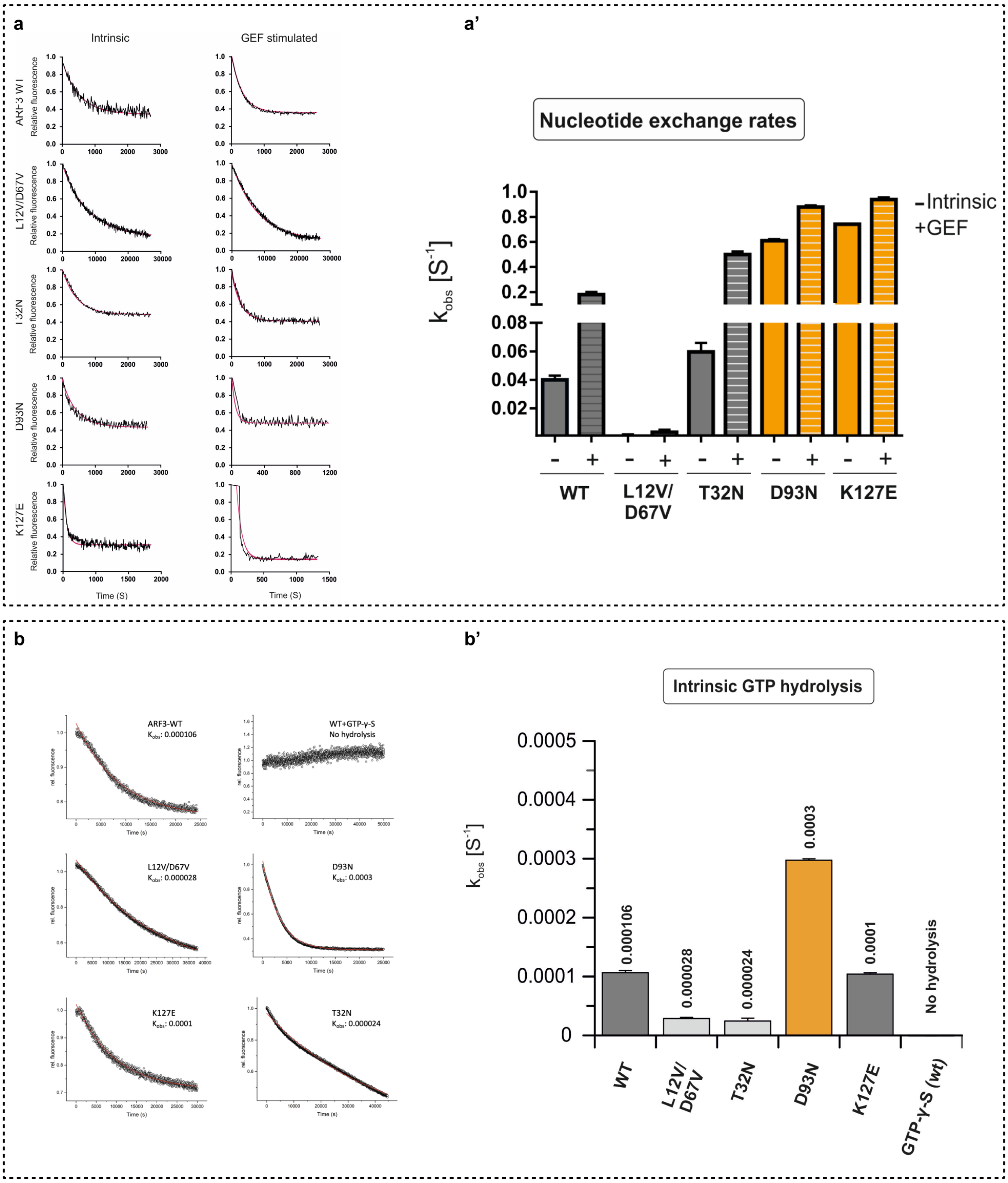

Supplementary Figure 6. Nucleotide exchange and GTP hydrolysis measurements of ARF3^{WT}, ARF3^{K127E}, ARF3$^{L12V/D67V}$, ARF3^{D93N} and ARF3^{T32N}. (**a**) Fluorescence polarization measurements showing the Intrinsic and GEF-mediated nucleotide exchange of the mantGDP release from which

the (k_{obs}) was derived (a) are shown in absence (left panel) or presence (right panel) of the SEC7 catalytic domain of ARFGEF BIG2. The y-axis display fluorescence polarization and the x-axis present time in second. (**a'**) Observed rate constants (k_{obs}) of intrinsic and GEF-mediated release of mantGDP for the indicated subsets of ARF3 purified proteins. The SEC7 catalytic domain of ARFGEF BIG2 was used as a ARF3GEF. The "–" bars represent the intrinsic nucleotide exchange and "+" bars represent GEF-mediated nucleotide exchange. (**b**) Fluorimeter measurements data using tamraGTP as a fluorescent reporter group plotted in b are shown. (**b'**) Observed rate constants (k_{obs}) of intrinsic GTP hydrolysis for the indicated subsets of ARF3 purified proteins. TamraGTP was rapidly mixed with the nucleotide-free $ARF3^{WT}$ in Cuvette in Fluorimeter and the GTP hydrolysis rate was measured as indicated above the bars. As a negative control, no hydrolysis reaction was detected for GTP-γ-S bound ARF3 wildtype. In a' and b' data are expressed as mean ± SD of independent experiments. Source data are provided as a Source Data file.

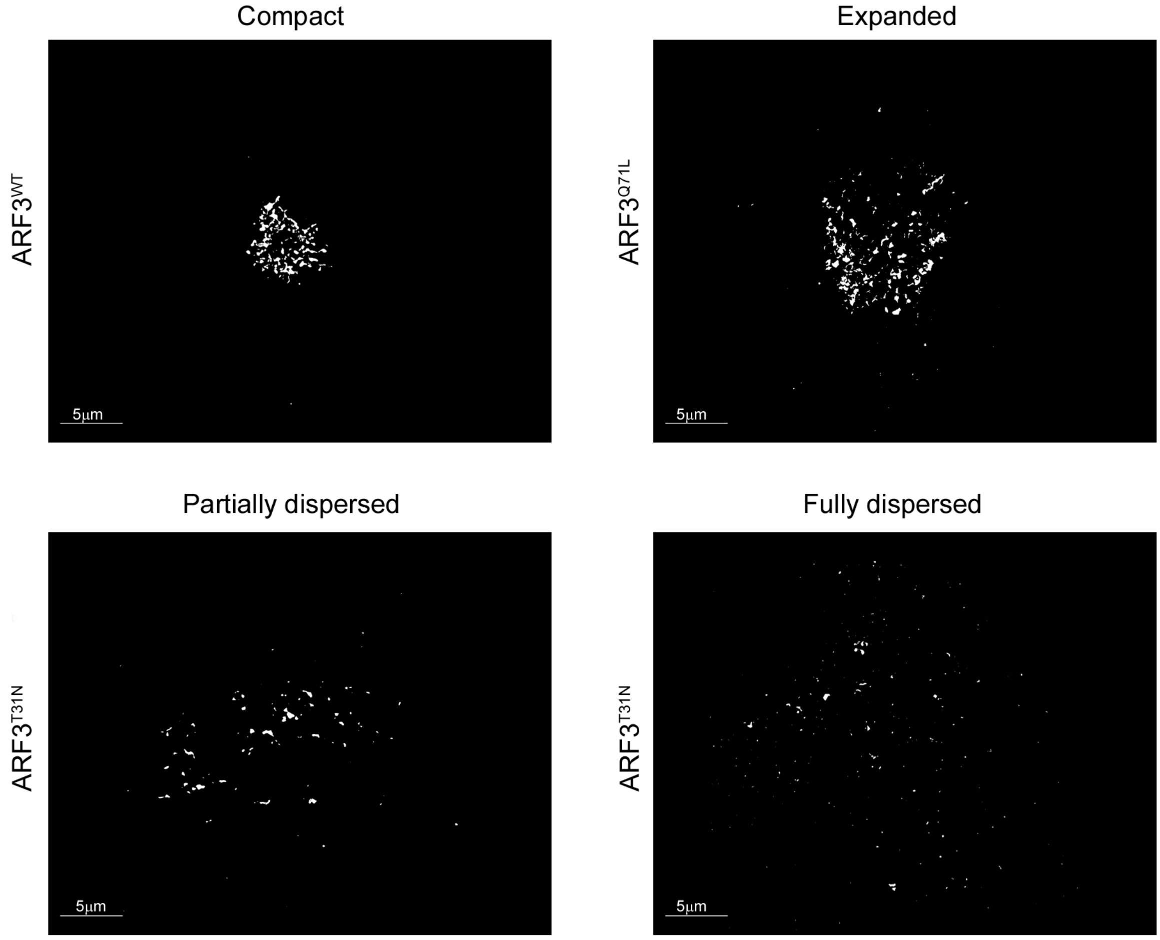

Supplementary Figure 7. 3D rendering of Golgi morphotypes observed in cells expressing ARF3^{WT}, ARF3^{Q71L} and ARF3^{T31N}, relative to Figure 3. 3D image reconstructions from confocal microscopy z-stack acquisitions of Golgin-97^{+} cisternae and vesicles (white) in COS1 cells transfected with mCherry-tagged ARF3^{WT}, or ARF3^{Q71L} and ARF3^{T31N} mutants. Scale bar = 5 μm.

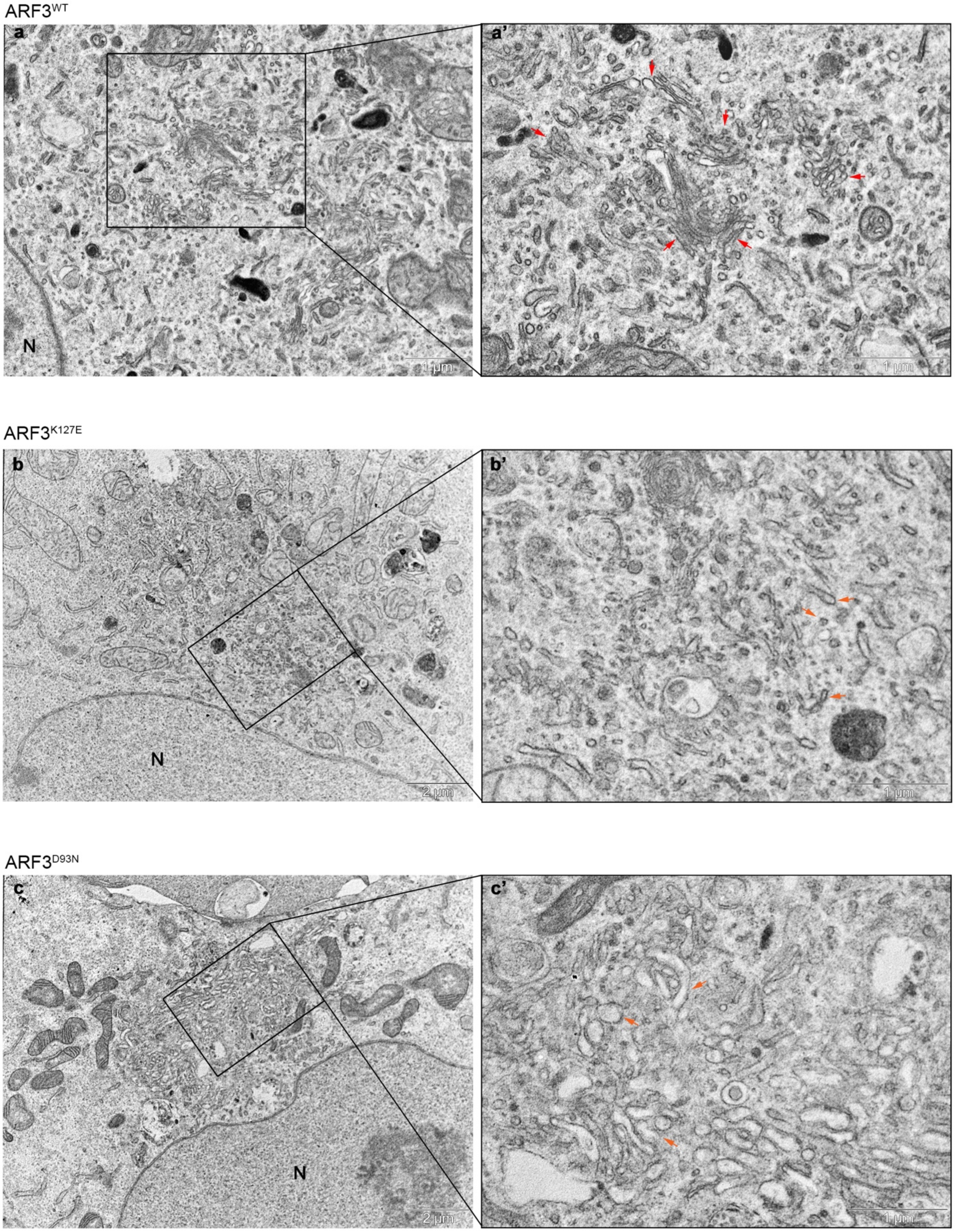
ARF3WT
a
a'
N
1 μm
1 μm
ARF3K127E
b
b'
N
2 μm
1 μm
ARF3D93N
c
c'
N
2 μm
1 μm

Supplementary Figure 8. Ultrastructural morphology of Golgi apparatus in COS-1 cells transfected with ARF3^{WT}, ARF3^{K127E} and ARF3^{D93N} revealed by TEM imaging. TEM images at different magnifications are shown for all the conditions examined over two independent experiments. (**a,a'**) Regular Golgi mini-stacks and ribbons are present in cells expressing ARF3^{WT}. Scale bar: 1 μm. (**b-c'**) different Golgi fragmentation patters and loss of integrity of the mini-stacks are observed in the ultrathin sections of cells expressing ARF3^{K127E} (b and high magnification of the squared area in b', scale bar: 2 and 1 μm, respectively) and ARF3^{D93N} (c and high magnification of the squared area in c', scale bar: 2 and 1 μm, respectively). "N" indicates the nucleus. Red arrows indicate normal Golgi mini-stacks and orange arrows indicate alterations in Golgi morphology in ARF3 mutants.

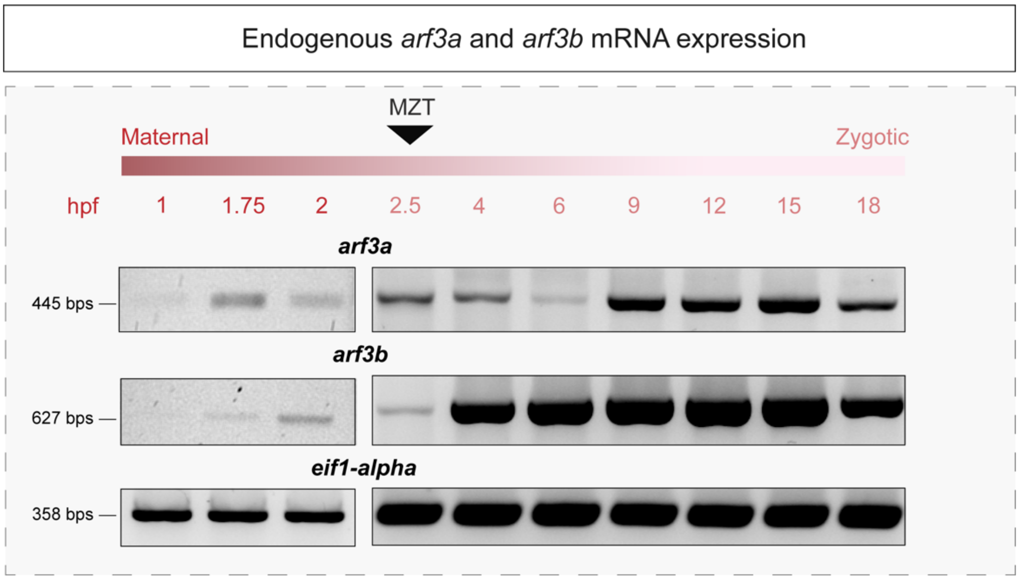

Supplementary Figure 9. Endogenous mRNA expression of *arf3a* and *arf3b* paralogs during early zebrafish embryogenesis. RT-PCR of the mRNA encoding arf3a and arf3b paralogs in zebrafish embryos from 1 to 18 hpf shows the maternal mRNA expression already from 1 hpf (*arf3a*) and 1.75 hpf (*arf3b*) as well as zygotic mRNA expression following MZT (about 2.5 hpf). Maternal *arf3a* and *arf3b* transcripts reach the highest expression from 9 hpf (segmentation) and 4hpf (gastrulation), respectively. *Eif1-alpha* is used as housekeeping control. The image shows the result of a single RT-PCR experiment.

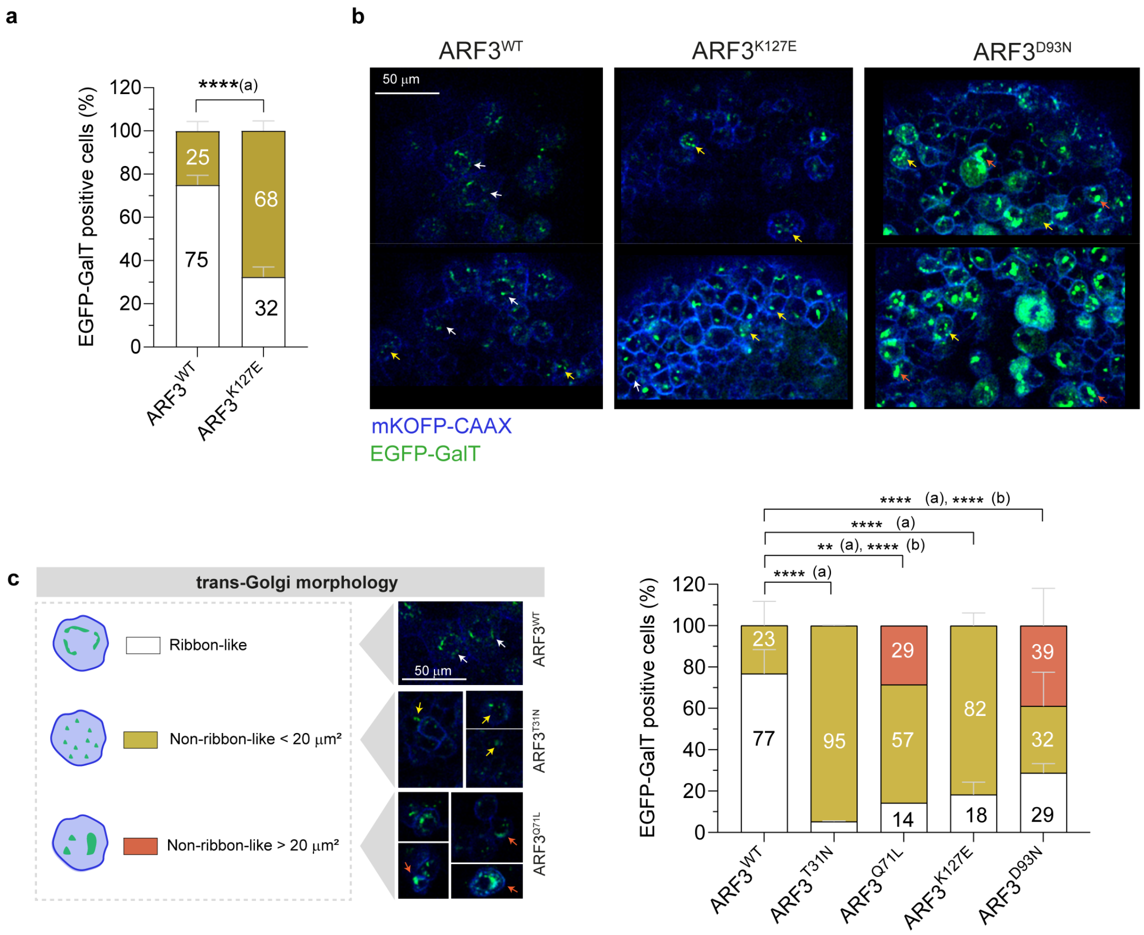

Supplementary Figure 10. Quantification of *trans*-Golgi morphology in precursor cells of the animal pole of 6 hpf zebrafish embryos expressing $ARF3^{WT}$, $ARF3^{K127E}$ and $ARF3^{D93N}$. (**a**) Incidence of the "ribbon-like" vs. "non-ribbon-like" phenotypes of EGFP-$GalT^+$ cells indicate *trans*-Golgi (TG) fragmentation in embryos from one batch overexpressing $ARF3^{K127E}$ compared to WT controls (referred to the confocal images from the main Fig. 4. 130 cells from n= 4 embryos (WT) and 174 cells from n= 5 embryos (K127E, ****p<0.0001). (**b**) Confocal images showing the *in vivo* staining of *trans*-Golgi (TG) marker (EGFP-GalT, magenta) and membrane marker (mKOFP, cyan) in cells of representative live embryos from one batch overexpressing ARF3 WT and mutant proteins (K127E and D93N) at the mid-gastrulation stage (~ 6 hpf). White arrowheads indicate a compact TG morphology surrounding the nucleus ("ribbon") in the envelope layer cells, yellow arrowheads indicate cells showing "*punta"* morphology of the TG dispersed throughout the cytosol. Scale bar = 50µm. (**c**) Schematics showing the TG (EGFP-$GalT^+$) phenotype classification, scored as "ribbon-like", "non-ribbon-like < 20 um^2" and "non-ribbon-like > 20 um^2" structures and incidence of cells showing different TG alterations among ARF3 mutants compared to WT controls. The reference images (for Q71L and T31N) are representative of cells from embryos of one batch. 46 cells (WT, $^{(a)}$) and 38 (T31N, ****p<0.0001$^{(a)}$) cells from n = 2 embryos; 7 cells from n = 1 embryo (Q71L,

**p=0.0056[(a)], ****p<0.0001[(b)]); 96 (K127E, ****p<0.0001[(a)]) and 134 (D93N, ****p<0.0001[(a, b)]) cells from n = 3 embryos. Data are expressed as mean ± SEM. Two sided Chi Square's test in 2x2 contingency table ([(a)] "ribbon-like structures" *vs.* "non-ribbon-like < 20 um^2", [(b)] "ribbon-like structures" *vs.* "non-ribbon-like > 20 um^2") is used for assessing statistical significance. Source data are provided as a Source Data file.

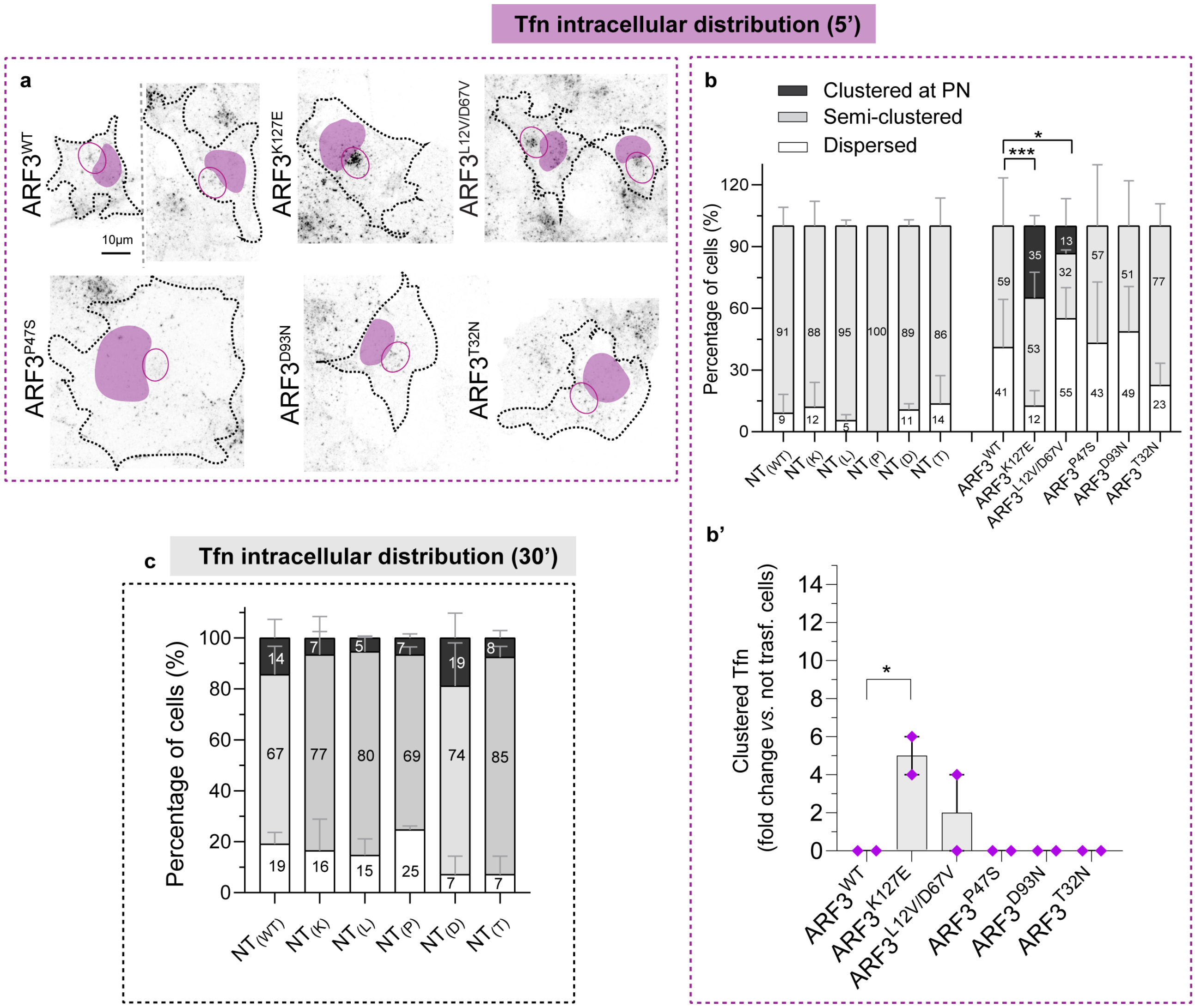

Supplementary figure 11. Intracellular Tfn distribution after 5 and 30 minutes of incubation. (**a**) Confocal maximum intensity z-projections showing the distribution of Tfn 488 (black) upon 5 min of incubation in COS-1 cells expressing WT and mutant ARF3. The black and white images are rendered by inverting the original LUT in Fiji. Red arrows indicate clustering of Tfn at the perinuclear region (PN, red circle). Nuclei are pseudo-colored in magenta. Images are representative of two independent experiments. (**b-b'**) Incidence of non-transfected control cells (NT) and cells transfected with all ARF3 mutants showing "clustered", "semi-clustered" or "dispersed" Tfn staining at 5 min **(b)**

and the ratio of the cells expressing ARF3 (WT and mutants) showing “clustered” Tfn phenotype normalized by the % of NT cells showing the same phenotype within the same slide preparation (b’, internal control). N of cells = 18 for NT (WT), 33 for NT (K127E), 63 for NT (L12V/D67V), 22 for NT (P47S), 35 (D93N), 26 for NT (T32N), 31 (ARF3WT), 30 (K127E, ***p=0.004), 25 (L12V/D67V, *p=0.0208), 32 (P47S), 37 (D93N), 26 (T32N). Data are expressed as mean ± SEM of two independent experiments (b and b’). (**c**) Incidence of non-transfected control cells (NT) showing the above-mentioned phenotypes at 30 min (relative to the main Fig. 6). N of cells = 69 for NT (WT), 101 for NT (K127E), 74 for NT (L12V/D67V), 100 for NT (P47S), 29 for NT (D93N), 25 for NT (T32N). Data are expressed as mean ± SEM of three (WT) and two (all mutants) independent experiments. Two-sided Chi square’s test in 2x2 contingency table (semi-clustered and dispersed vs, clustered, b and c) or One-way ANOVA with Dunnett’s multiple comparison *post hoc* test (b’) are used. Source data are provided as a Source Data file.

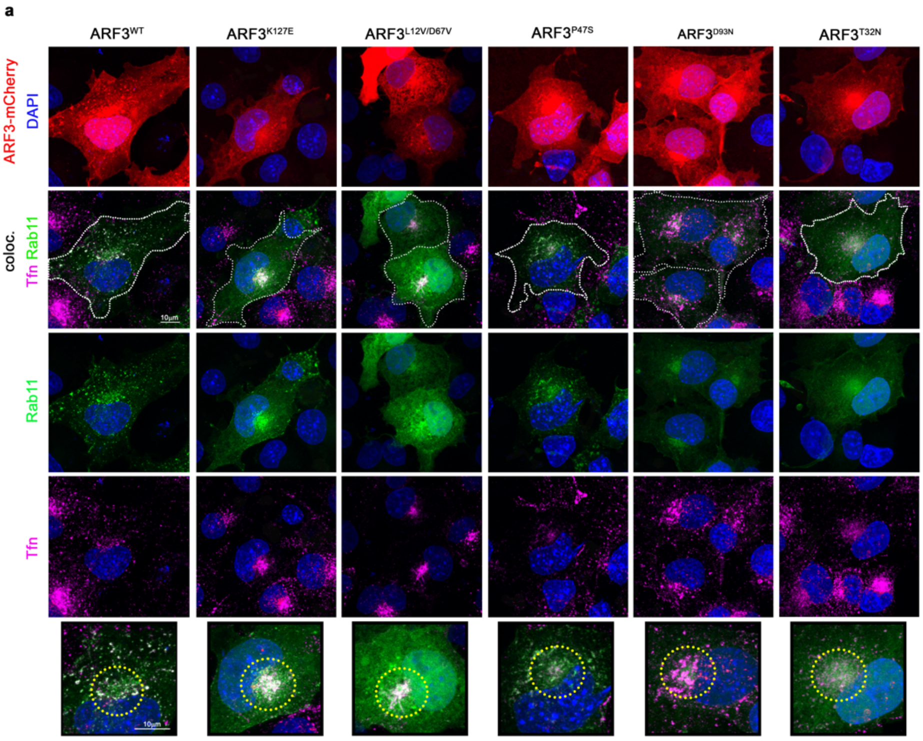

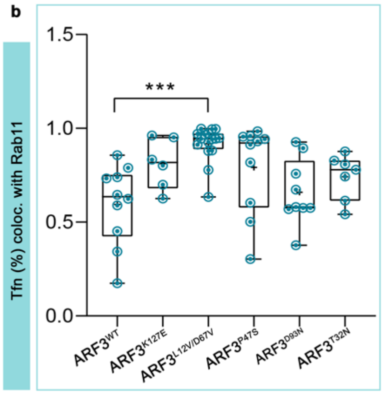

Supplementary Figure 12. Co-localization analysis of Tfn and Rab11 at PN upon 30 min of Tfn incubation. (**a**) Confocal maximum intensity z-projections showing images of COS-1 cells co-transfected with mCherry-tagged WT or mutant ARF3 and EGFP-Rab11 expression plasmids and incubated with Tfn 647 for 30 min. Rab11 marks recycling endosomes. Single channels and colocalization (merge) between Tfn and Rab11 are shown for each genetic condition (red: ARF3mCherry expression, green: Rab11 expression, magenta: intracellular Tfn staining, blue: nuclei stained with DAPI). Images are representative of cells from a single experiment. For each condition, a zoom on the perinuclear region (PN) used for the analysis is shown in the lower insets. Scale bar = 10 µm. (**b**) Co-localization analysis showing the % of Tfn^+ fluorescent signal co-occurring with $Rab11^+$ signal within the PN region as analyzed by thresholded Mander's coefficient M1. N of cells

= 10 (WT), 6 (K127E), 16 (L12V/D67V, ***p=0.0002), 10 (P47S), 9 (D93N) and 7 (T32N). Data are expressed as box-and-whisker with median (middle line), 25th–75th percentiles (box), and min-max values (whiskers). All the data points and the mean ("+") are also shown. Kruskal-Wallis with Dunn's *post hoc* test is used to assess statistical significance. Source data are provided as a Source Data file.

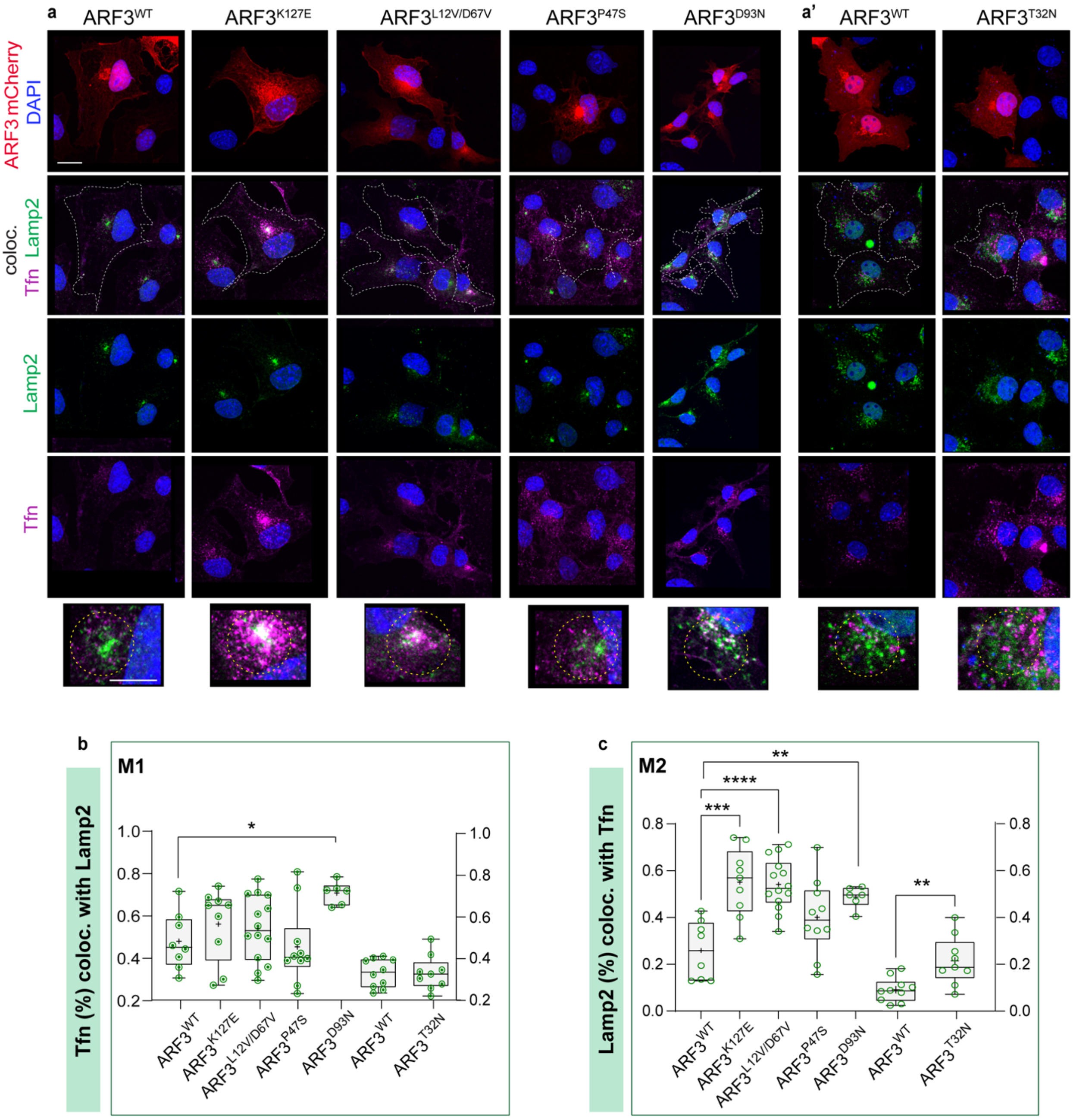

Supplementary Figure 13. Altered lysosomal trafficking of Tfn upon 30 min of incubation in cells expressing ARF3 mutations. (**a**) Confocal maximum intensity z-projections showing images of COS-1 cells expressing mCherry-tagged ARF3 WT and all the identified mutants immunostained for the lysosomal marker Lamp2 following 30 min stimulation with Tfn. Single channels and colocalization (merge) between Tfn and Lamp2 are shown for each genetic condition (red: ARF3mCherry expression, green: Lamp2 staining, magenta: intracellular Tfn staining, blue: nuclei

stained with DAPI). For each condition, a zoom on the perinuclear region (PN) used for the analysis is shown in the lower insets. Scale bar = 20 µm and 10 µm (upper panel and zoomed images, respectively). Images are representative of two independent experiments. (**b, c**) Colocalization analysis showing the % of Tfn^+ fluorescent signal co-occurring with $Lamp2^+$ signal within the PN region and *viceversa*, as analyzed by thresholded Mander's coefficients (M1 and M2). Different sets of data for the same measurement are shown in adjacent plots. The internal WT control is shown for each set. For set 1: n of cells = 8. K127E: ***p=0.001, L12V/D67V: ****p<0.0001, P47S, D93N: *p=0.033 in b and **p=0.0068 in c. For set 2: n of cells = 10 (^{WT}) and 9 (^{T32N}, **p=0.0039) from a single experiment. Data are expressed as box-and-whisker with median (middle line), 25th–75th percentiles (box), and min-max values (whiskers). All the data points and the mean ("+") are also shown. (b and c). Krustal-Wallis followed by Dunn's multiple comparison *post hoc* test or unpaired t-test are used for M1 analysis (b) or One-way ANOVA followed by Dunnett's multiple comparison *post hoc* test or unpaired t-test are used for M2 analysis (c). Source data are provided as a Source Data file.

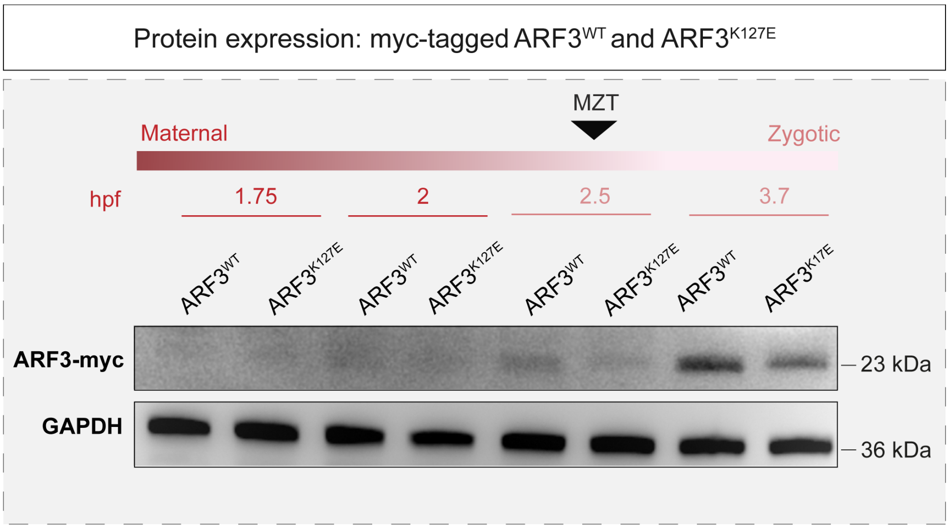

Supplementary Figure 14. Expression of myc-tagged ARF3 during early zebrafish embryogenesis. Western blot from total protein extracts of embryos injected with capped mRNA expressing myc-tagged $ARF3^{WT}$ and $ARF3^{K127E}$ shows that the expression is minimal in the early stages of embryogenesis (1 - 1.75 hpf) and increases from about 2.5 hpf (MZT). Lower expression of myc-tagged $ARF3^{K127E}$ is observed already at these early stages. GAPDH (reference gene, ~37 kDa), myc-tagged ARF3 (~23 kDa). The blot is representative of two independent experiments.

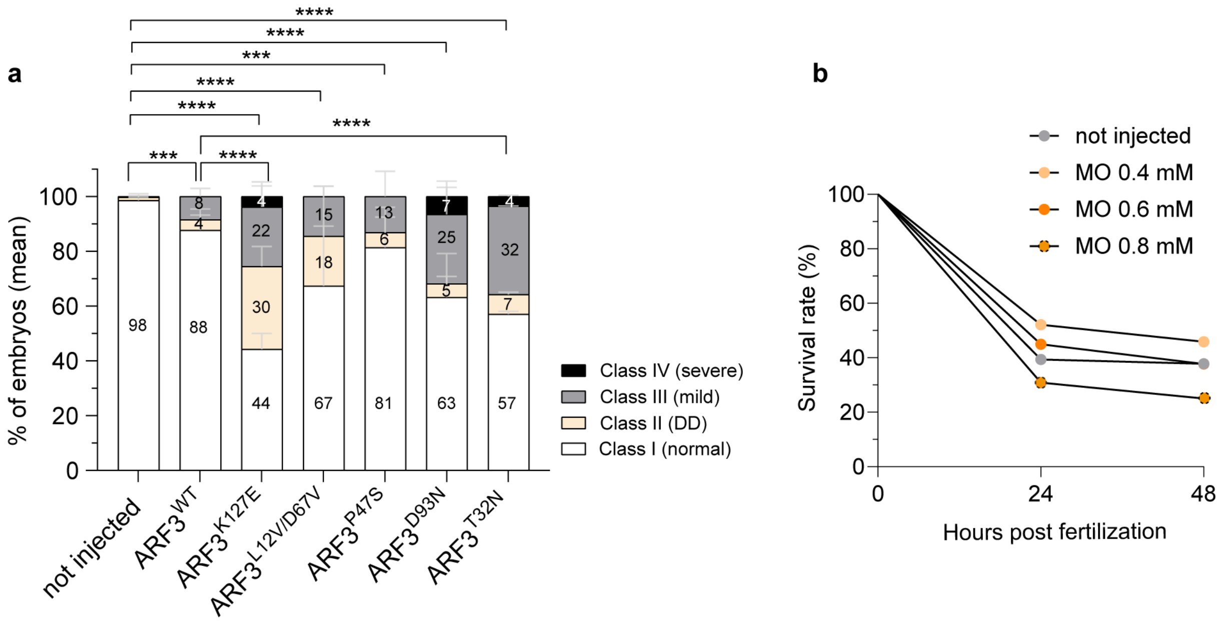

Supplementary Figure 15. Incidence of gross phenotypes in fish overexpressing WT and mutant ARF3 at 48 hpf and survival curve of *arf3a* and *arf3b* MO-injected fish at 24 and 48 hpf. (**a**) Incidence of gross phenotypes of fish injected with mRNA encoding all the identified ARF3 mutants, ARF3WT and not injected (not inj.) controls at 48 hpf (refer to main Fig. 7). By 48hpf also ARF3^{WT} induces a slight increase of class III features, indicating that a fine control of ARF3 function is required during development. N of embryos = 131 (not inj.), 67 (WT, ***p=0.0007), 21 (K127E, *vs.* not inj. or WT, ****p<0.0001) 59 (L12V/D67V, *vs.* not inj. ****p<0.0001) 43 (P47S, *vs.* not inj ***p=0.0006) 81 (D93N, *vs.* not inj. ****p<0.0001) 42 (T32N, *vs.* not inj. or WT ****p<0.0001). Data are expressed as mean ± SEM of four (not inj., WT), three (D93N) and two (K127E, L12V/D67V, P47S, T32N) batches. (**b**) Survival curve of MO-injected fish with different doses of *arf3a* and *arf3b* MOs, n of embryos = 127 (not inj.), 48 (MO 0.4 mM), 69 (MO 0.6 mM) and 68 (MO 0.8 mM) of one batch. Two-sided Chi-square test in 2x2 contingency table (global phenotype *vs.* normal) is used to assess statistical significance in a. Log-Rank test (Mantel-Cox) is used to assess survival in b. Source data are provided as a Source Data file.

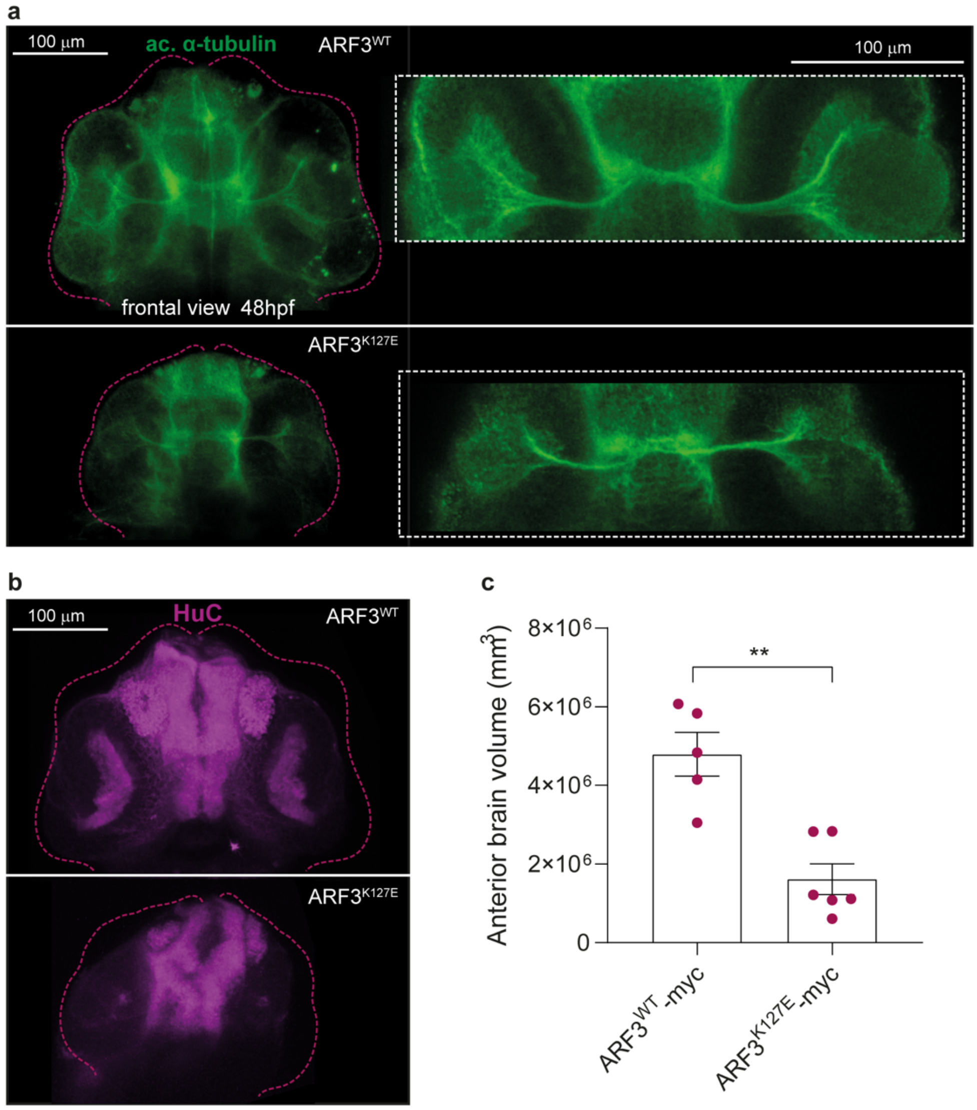

Supplementary Figure 16. Anterior brain volume at 48 hpf is reduced in zebrafish expressing ARF3^{K127E}. (**a**,**b**) Confocal maximum intensity z-projections of the anterior brain (outlined by purple dashed line) of 48 hpf fish injected with mRNA encoding ARF3^{WT} (up) and ARF3^{K127E} (bottom) and stained with antibodies against α-acetylated tubulin (green) or HuC/elav (magenta). On the upper right a close-up of a more ventral domain around the optic chiasm is shown. Images are representative of embryos from two batches. (**c**) Measurement of the anterior most brain volume (HuC/elav) of 48 hpf embryos expressing ARF3^{WT} and ARF3^{K127E}. N of embryos = 5 (WT) and 6 (K127E, **p=0.0019) of one batch. Data are expressed as mean ± SEM. Unpaired t-test followed by Welch's *post hoc* test). Source data are provided as a Source Data file.

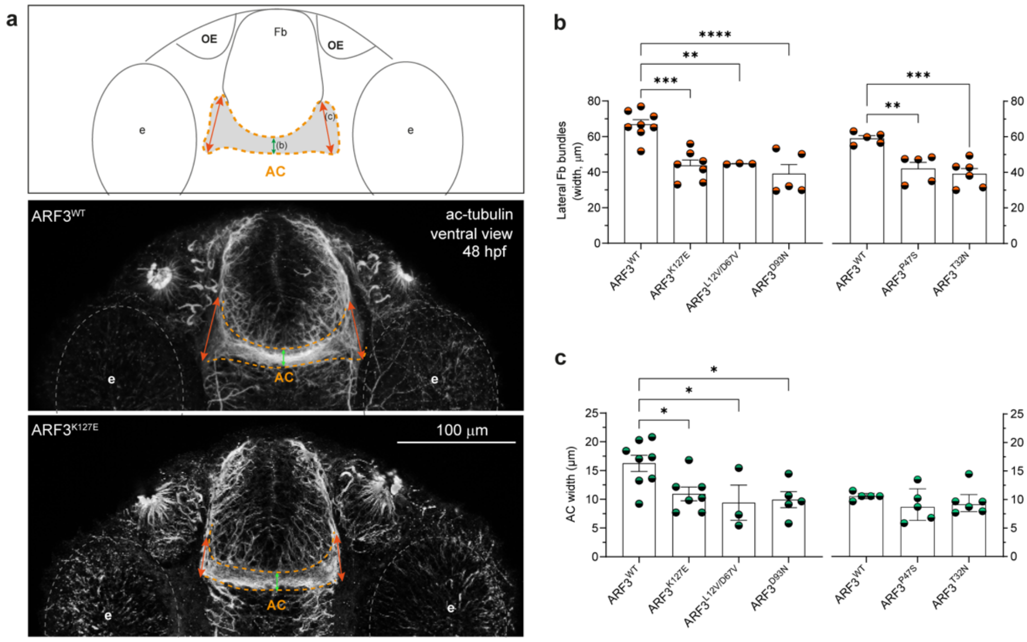

Supplementary figure 17. Zebrafish embryos expressing ARF3 mutants exhibit thinning of the major tract of the forebrain white matter. (**a**) Schematics of the anterior commissure (AC, orange) of the forebrain (Fb) observed from ventral view of the anterior zebrafish brain at 48 hpf (upper panel) and confocal z-projections (standard deviation) of the ventral anterior brain showing the axonal scaffold labeled with antibody against acetylated tubulin in $ARF3^{WT}$ and $ARF3^{K127E}$-overexpressing zebrafish embryos (lower two panels). The images are representative of embryos from one batch. (**b, c**) Quantification of the width of lateral Fb bundles (b) and medial AC width (c). The two parameters measured are depicted by green and dark orange arrows, respectively, in the scheme in a and in the confocal scans. Scale bar = 100 µm. OE: olfactory epithelium, e: eye. Set 1: n of embryos = 8 (WT), 7 (K127E, ***p=0.0002 for b, *p=0.035 for c), 3 (L12V/D67V, **p=0.0034 for b, *p=0.0381 for c), 5 (D93N, **** p<0.0001 for b, *p=0.0219 for c), set 2: 5 (WT), 5 (P47S, **p=0.0024 for b) and 6 (T32N, ***p=0.0005 for b) of one batch. Data are shown as mean ± SEM (for b and the left graph in c) and median with interquartile range (c, right). Different datasets for the same measurement are shown in adjacent plots with the internal WT control for each set. One-way ANOVA followed by Dunnett's multiple comparison *post hoc* test is used (b, left and right and c, left) or Kruskal-Wallis followed by Dunn's multiple comparison *post hoc* test are used (c, right) are used to assess statistical significance. Source data are provided as a Source Data file.

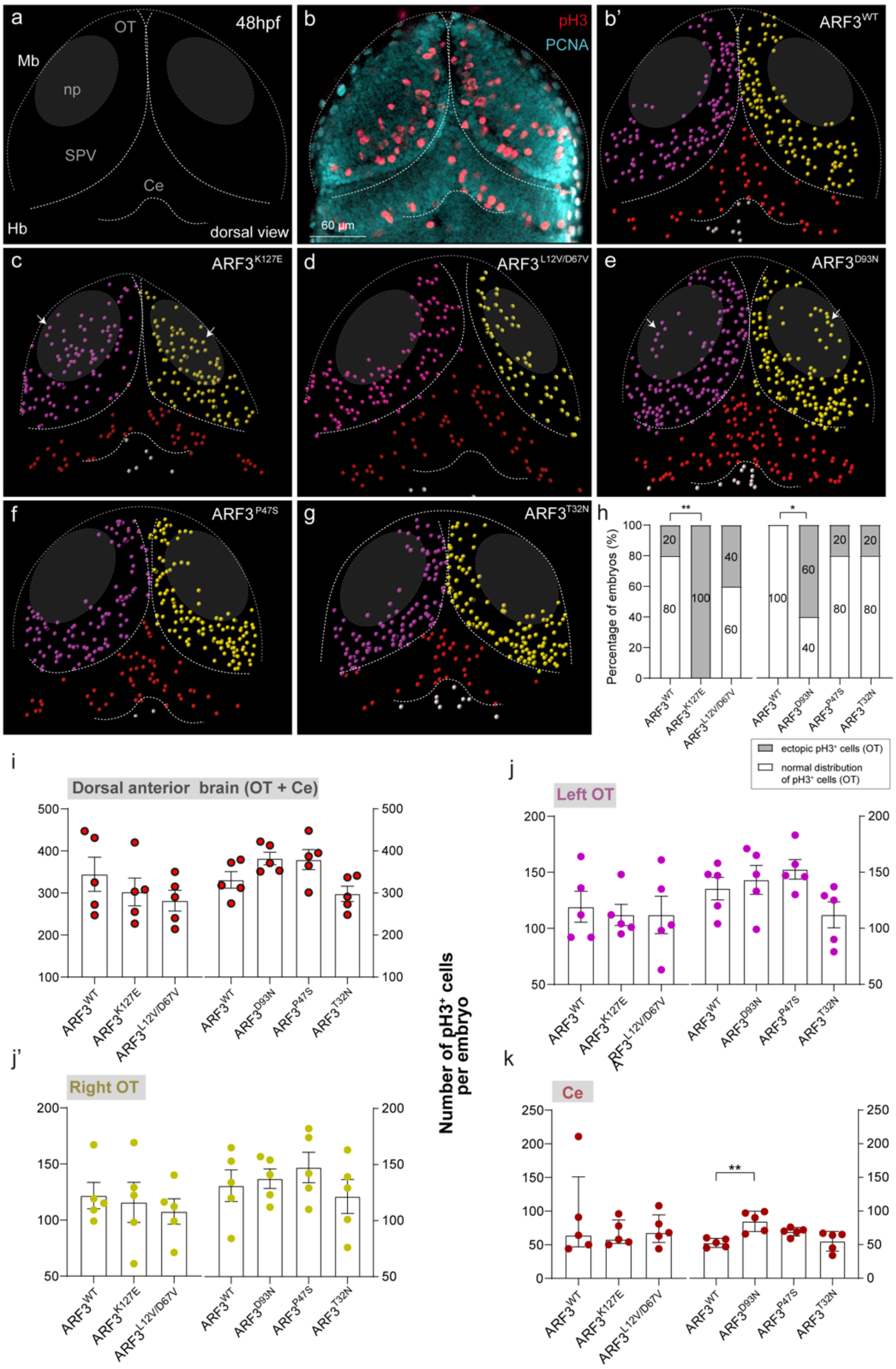

Supplementary Figure 18. Ectopic proliferative cells are found within the developing diencephalon of zebrafish expressing ARF3^{K127E} and ARF3^{D93N} mutants. (**a**) Schematic overview of the dorsal anterior brain (midbrain, Mb and hindbrain, Hb) in developing zebrafish embryos at 48hpf. OT: optic tectum, NP: optic tectum neuropil, SPV: *stratum periventricolare*, Ce: cerebellum. The outline of the OT and Ce are shown with dashed white line, the NP is indicated by grey circle. (**b**) Confocal maximum intensity z-projection showing proliferative and mitotic cells in the midbrain and anterior hindbrain of zebrafish embryos from one batch overexpressing ARF3^{WT}. The cells are

stained with antibodies against PCNA and phospho-histone 3 (pH3). The images are representative of embryos from one batch. (**b'-g**) Segmented datasets obtained from confocal 3D scans, reconstructing the proliferative $pH3^+$ cells within the dorsal anterior brain of zebrafish expressing $ARF3^{WT}$ and all the identified ARF3 mutants. Each spot represents a pH3+ cell. Cells within different domains are pseudo-labeled with different colors (purple and yellow for cells within the left and right OT, respectively, red for cells within the Ce). (**h**) Incidence of embryos expressing $ARF3^{WT}$ and all the mutants showing ectopic $pH3^+$ within the OT (cells wrongly localized in the NP territory, white arrows in c and e). N of embryos = 5, (K127E **p = 0.0098 and D93N *p = 0.0384) of one batch. (**i-k**) Quantification of the number of $pH3^+$ cells labeled within the dorsal anterior brain: total number (i), cells found in the left OT (j) or right OT (j') and in the Ce (k). N of embryos = 5. ($ARF3^{D93N}$ **p = 0.0012) of one batch. Data are shown as mean ± SEM (I, j-j', k right) or median with interquartile range (k, left). Different datasets for the same measurement are shown in adjacent plots with the internal WT control for each set. Two-sided Chi-square's test in 2x2 contingency table (h), One-way ANOVA followed by Dunnett's multiple comparison *post hoc* test (j-j', k right) or Kruskal-Wallis followed by Dunn's multiple comparison *post hoc* test (k, left) are used to assess statistical significance. Source data are provided as a Source Data file.

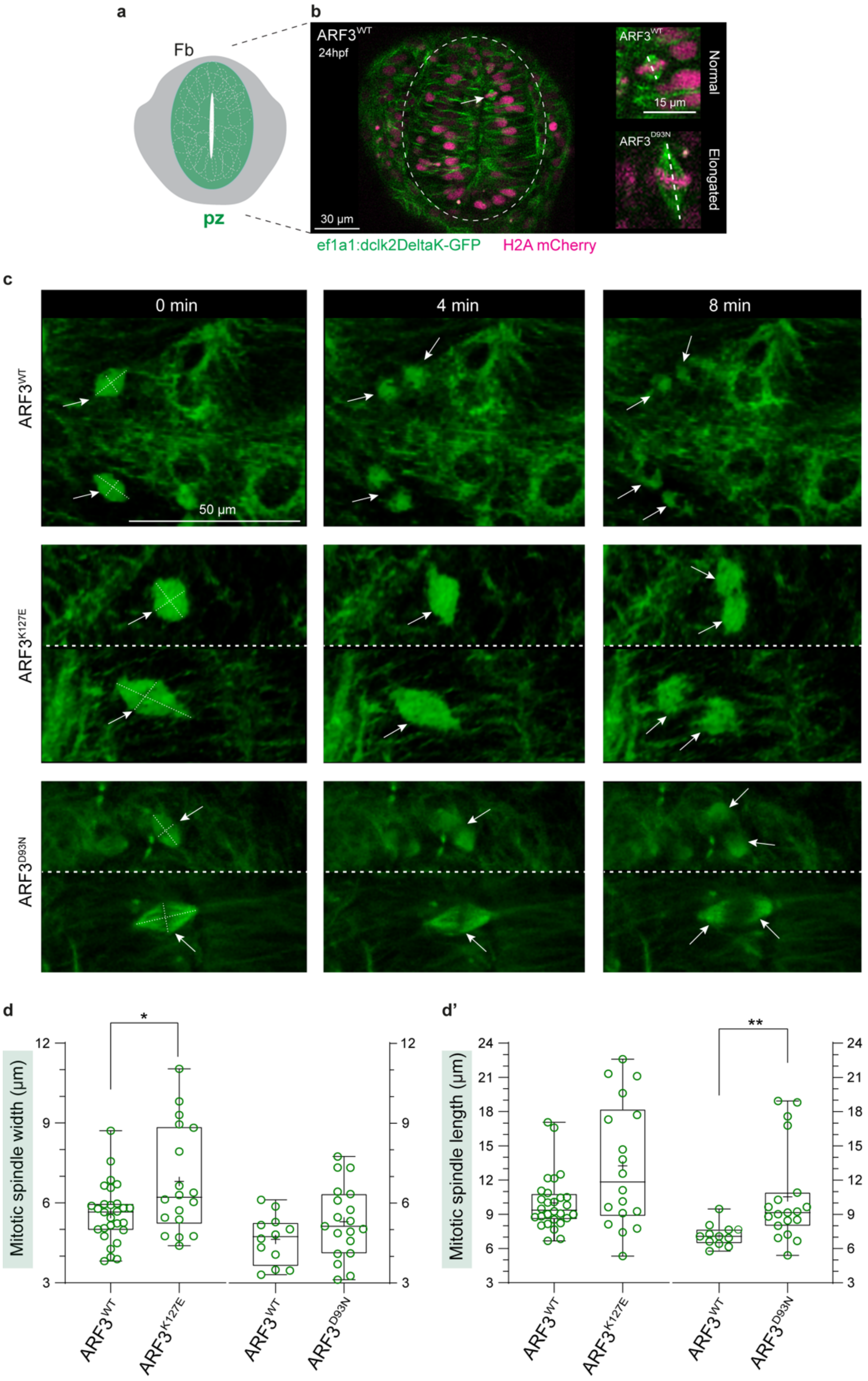

Supplementary Figure 19. Impaired spindle morphology of mitotic cells within the developing forebrain of zebrafish embryos expressing ARF3^{K127E} and ARF3^{D93N}. (**a**) Schematics depicting the zebrafish proliferative zone (pz) of the forebrain (Fb) imaged at ~ 28hpf. (**b**) Confocal maximum intensity z-projection showing the Fb pz of a live embryo injected with mRNA encoding ARF3^{WT} and H2AmCherry (chromatin marker) in live transgenic fish *Tg(XlEef1a1:dclk2DeltaK-GFP)* in which the

tubulin marker dclk2 labels the polymerized microtubules. (**b'**) Close-ups on confocal z-scans of the Fb pz of fish injected with ARF3^{WT} (cell indicated by white arrow in b) and ARF3^{D93N} showcase the elongated spindle of metaphasic cells in mutant forebrain. (**c)** Close-ups on confocal z-scans showing diving cells during the time lapse for the mutants and WT controls. Images are representative of embryos from one batch. White arrows indicate cell cycle progression from metaphase to anaphase/telophase. Time lapses (50 min in total) were performed consecutively for ARF3^{WT} and ARF3^{D93N} and were simultaneous (for 16h in total) for ARF3^{WT} and ARF3^{K127E}. (**d-d'**) Quantification of the width and length of the mitotic spindle of cells (as shown by dashed white line in c) within the brain for both mutants and WT controls, set 1: n of cells = 28 (WT), 18 (K127E, *p = 0.0118 in d) from one embryo each, set 2: n of cells = 12 (WT) and 19 (D93N, **p = 0.0022 in d') of one embryo each. Different datasets for the same measurement are shown in adjacent plots with the internal WT control for each set. Data are expressed as box-and-whisker with median (middle line), 25th–75th percentiles (box), and min-max values (whiskers). All the data points and the mean value ("+") are also shown. Two-tailed Unpaired T-Student test (d) and non-parametric Mann-Whitney (d') are used to assess statistical significance. Source data are provided as a Source Data file.

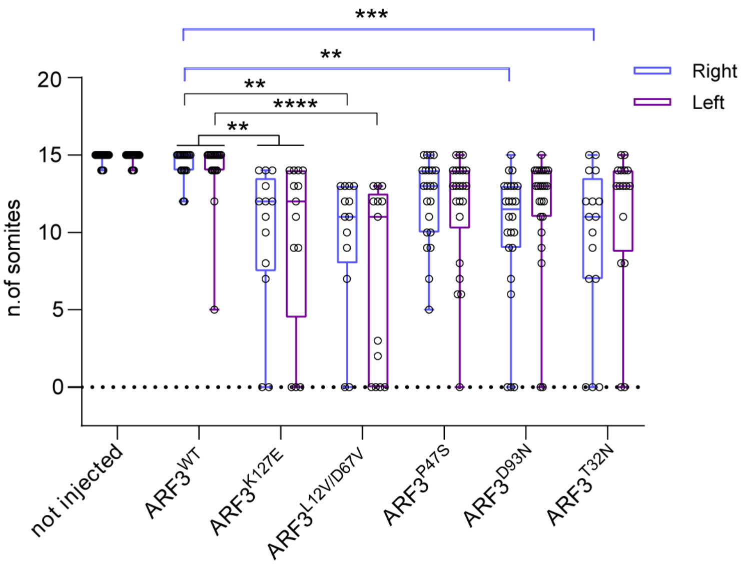

Supplementary Figure 20. Assessment of the number of somites in ARF3 mutants at 15 hpf. The total number of visible *MyoD*$^{+}$ somites on the left and right side of the embryos is plotted. N of embryos = 24 (not injected), 18 (WT), 13 (K127E, right, **p=0.0053, left, **p=0.0065), 13 (L12V/D67V, right, **p=0.0029, left, ****p<0.0001), 22 (P47S), 24 (D93N, right, ** p=0.0012), 16 (T32N, right, ***p=0.0007) of one batch. Data are shown as box-and-whisker with median (middle line), 25th–75th percentiles (box), and min-max values (whiskers). All the data points and the mean

("+") are also shown. Two-way ANOVA with Sidak's *post hoc* test, with mixed-effects model analysis (2 categories: «Left and Right» and «genetic condition») is used to assess statistical significance. Source data are provided as a Source Data file.

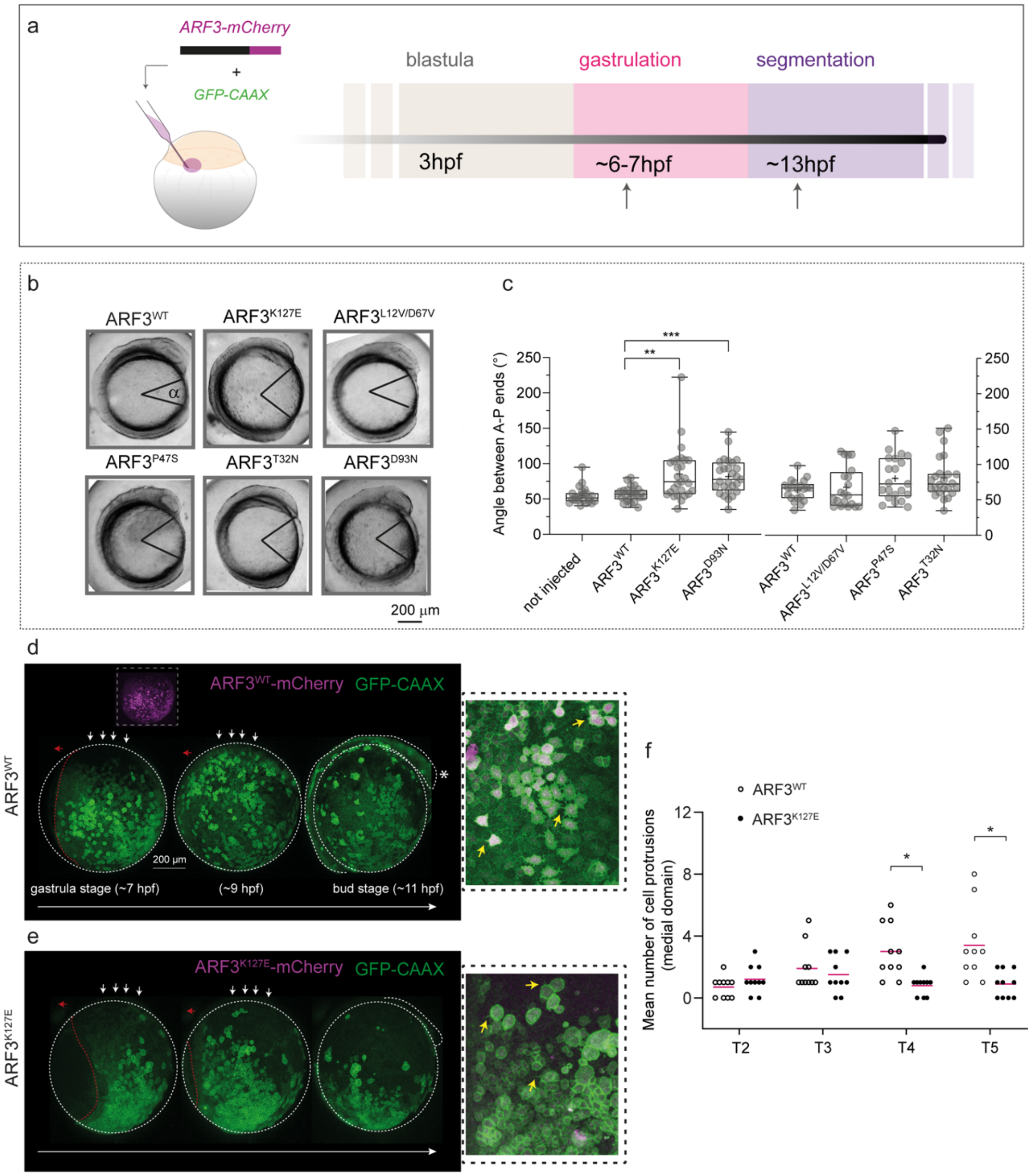

Supplementary Figure 21. Overexpression of ARF3^{K127E} and ARF3^{D93N} in zebrafish embryos impairs convergence and extension movements during gastrulation. (**a**) Schematics of the experimental design. mCherry-tagged ARF3 and GFP-tagged CAAX are injected in one-cell stage embryos and morphometric measurements are performed between 6 hpf (gastrulation) and 13 hpf (segmentation). (**b**) Bright field images from live embryos injected with mRNA encoding ARF3^{WT} and all the identified mutant ARF3 at 13 hpf. Lateral views with anterior cephalic region (developing head) on the top left are shown. The angle (α, black) between the anterior tip and the posterior end is shown. The images are representative of embryos from two independent batches. (**c**) Values of the α angles showing the antero-posterior (AP) extension in ARF3^{WT} and mutants are shown. Set 1: n of embryos = 23 (not injected), 24 (WT), 27 (K127E, **p=0.002) and 27 (D93N, ***p=0.0009), set 2: 21 (WT), 21 (L12V/D67V), 21 (P47) and 24 (T32N) of one batch. Data are expressed as box-and-whisker with median (middle line), 25th–75th percentiles (box), and min-max values (whiskers). All the data points and the mean value ("+") are also shown. Different sets of data for the same measurement are shown in adjacent plots. The internal WT control is shown for each set. (**d-e**) Confocal images (maximum z-projections) from a single live time-lapse experiment showing lateral view of the embryos at different time points during gastrulation. Images show cells labelled with GFP-tagged CAAX marking cellular outlines. Expression of mCherry-tagged ARF3^{WT} in the injected embryos is shown in the upper inset. Higher magnifications on the right (black dashed boxes) show cell protrusions (some are indicated by yellow arrows) of medial cells at ~ 8 hpf in ARF3^{WT} and ARF3^{K127E} embryos. (**f**) Number of cell protrusions counted in the medial portion (centre) of each embryo (WT and K127E) between 7 and 8 hpf. N of cells = 10 (WT and K127E, *p=0.0131 at T4, *p=0.0360 at T5). In the scatter plot dots represent the mean number of cell protusions per cell and the purple line indicates the mean value of the cells counted in one embryo per each condition. Non-parametric Kruskal-Wallis followed by Dunn's multiple comparison *post hoc* test (c) or Two-way ANOVA with Sidak's *post hoc* test, with mixed-effects model analysis to control for resampling from the same cluster (more cells from one embryo) followed by Sidak's multiple comparison *post hoc* test (f) are used to assess statistical significance. Source data are provided as a Source Data file.

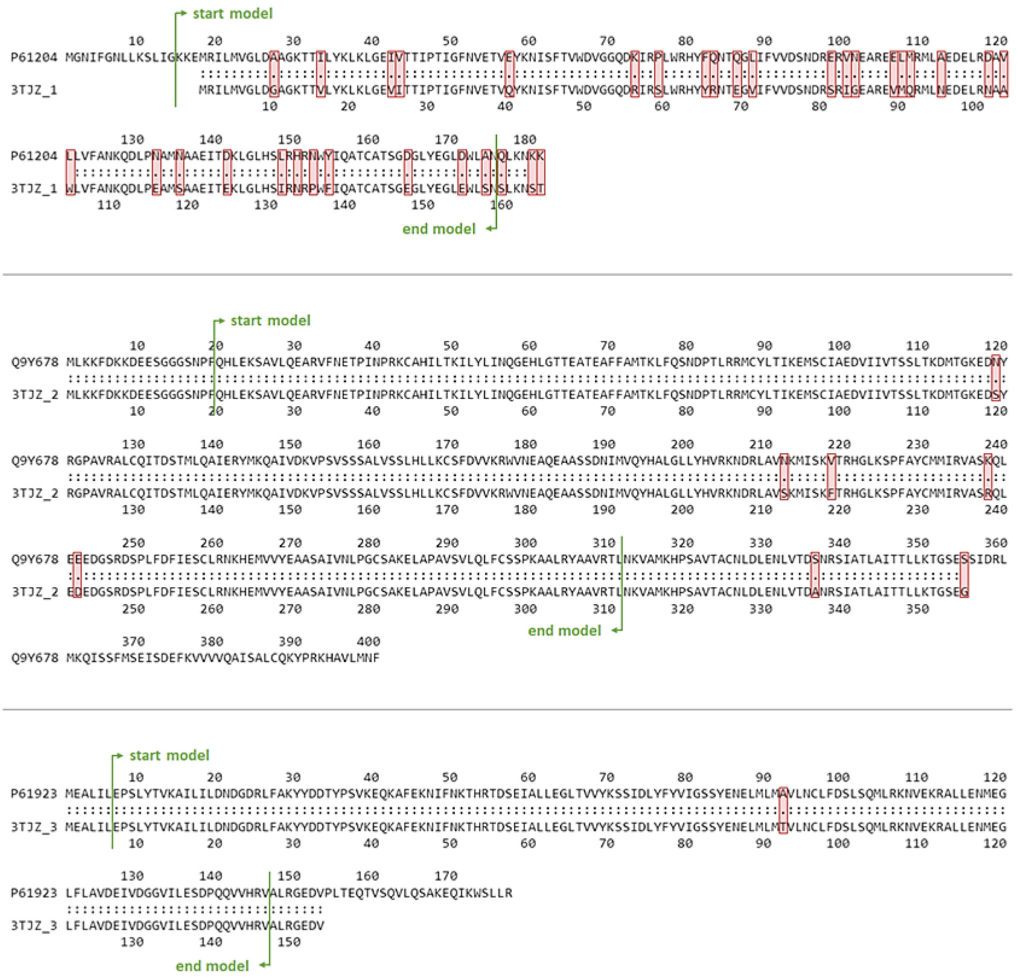

Supplementary Figure 22. Comparison of the FASTA sequences available in the UniProt database and the PDB entry 3TJZ. The panel above aligns the amino acid sequence of ADP-ribosylation factor 3 (ARF3) from *Homo sapiens* (identifier: P61204-1) and ADP-ribosylation factor 1 (ARF1) from *Saccharomyces cerevisiae* (3TJZ_1). The middle panel compares the amino acid sequence of Coatomer subunit gamma-1 (COPG1) from *Homo sapiens* (identifier: Q9Y678) and COPG1 from *Bos taurus* (3TJZ_2). The panel below confronts the amino acid sequence of Coatomer subunit zeta-1 (COPZ1) from *Homo sapiens* (identifier: P61923) and COPZ1 from *Bos taurus* (3TJZ_3). The red rectangles highlight the differences between the sequences and the green markings identify the beginning and end of the model based on the UniProt sequence and the 3TJZ PDB 3D structure.

Supplementary References

1. Amadei, A., Linssen, A.B., Berendsen, H.J. (1993). Essential dynamics of proteins. *Proteins* 17, 412–425.

2. Bauer, C.K., Calligari, P., Radio, F.C., *et al*. (2018). Mutations in KCNK4 that Affect Gating Cause a Recognizable Neurodevelopmental Syndrome. *Am J Hum Genet* 103, 621-630.
3. Cingolani P, Sladek R, Blanchette M. (2015) BigDataScript: a scripting language for data pipelines. *Bioinformatics* 31, 10-16.
4. Cooper G.M., Stone E.A., Asimenos G., *et al*. (2005) Distribution and intensity of constraint in mammalian genomic sequence. *Genome Res* 15, 901-913.
5. DePristo M.A., Banks E., Poplin R., *et al*. (2011) A framework for variation discovery and genotyping using next-generation DNA sequencing data. *Nat Genet* 43, 491-498.
6. Iascone M., Sana M.E., Pezzoli L., *et al*. (2012) Extensive arterial tortuosity and severe aortic dilation in a newborn with an EFEMP2 mutation. *Circulation* 126, 2764-2768.
7. Jagadeesh KA, Wenger AM, Berger MJ, *et al*. (2016) M-CAP eliminates a majority of variants of uncertain significance in clinical exomes at high sensitivity. *Nat Genet* 48, 1581-1586.
8. Kircher M, Witten DM, Jain P, *et al*. (2014) A general framework for estimating the relative patho-genicity of human genetic variants. *Nat Genet* 46, 310-315.
9. Li Q, Wang K. (2017) InterVar: Clinical Interpretation of Genetic Variants by the 2015 ACMG-AMP Guidelines. *Am J Hum Genet* 100, 267-280.
10. Li H. (2013) Aligning sequence reads, clone sequences and assembly contigs with BWA-MEM. *arXiv*, arXiv:1303.3997.
11. Li H, Durbin R. (2009) Fast and accurate short read alignment with Burrows-Wheeler transform. *Bioinformatics* 25, 1754-1760.
12. Liu X., Jian X., Boerwinkle E. (2011) dbNSFP: a lightweight database of human nonsynonymous SNPs and their functional predictions. *Hum Mutat* 32, 894-899.
13. Liu X, Jian X, Boerwinkle E. (2013) dbNSFP v2.0: a database of human non-synonymous SNVs and their functional predictions and annotations. *Hum Mutat* 34, E2393-23402.
14. Motta, M., Pannone, L., Pantaleoni, F., *et al*. (2020). Enhanced MAPK1 Function Causes a Neurodevelopmental Disorder within the RASopathy Clinical Spectrum. *Am J Hum Genet* 107, 499-513.
15. Pezzani L., Marchetti D., Cereda A., *et al*. (2018) Atypical presentation of pediatric BRAF RASopathy with acute encephalopathy. *Am J Med Genet A* 176A, 2867-2871.
16. Radio F.C., Pang K., Ciolfi A., *et al*. (2021) SPEN haploinsufficiency causes a neurodevelopmental disorder overlapping proximal 1p36 deletion syndrome with an episignature of X chromosomes in females. *Am J Hum Genet* 108, 502-516.
17. Shi Y., Mowery R.A., Ashley J., *et al*. (2012) Abnormal SDS-PAGE migration of cytosolic proteins can identify domains and mechanisms that control surfactant binding. *Protein Sci. Publ. Protein Soc*. 21, 1197–1209.

18. Thorvaldsdóttir H., Robinson J.T., Mesirov J.P. (2013) Integrative Genomics Viewer (IGV): high-performance genomics data visualization and exploration. *Brief Bioinform* 14, 178-192.
19. Van der Auwera G.A., Carneiro M., Hartl C., *et al*. (2013) From FastQ Data to High-Confidence Variant Calls: The Genome Analy-sis Toolkit Best Practices Pipeline. *Curr Protoc Bioinformatics* 43, 11.10.1-11.10.33.
20. Vetro A., Nielsen H.N., Holm R., *et al*. (2021) Vetro ATP1A2- and ATP1A3-associated early profound epileptic encephalopathy and polymicrogyria. Brain, doi: 10.1093/brain/awab052.

Uncropped blots and gels relative to the supplementary figures

Suppl.Figure 4a

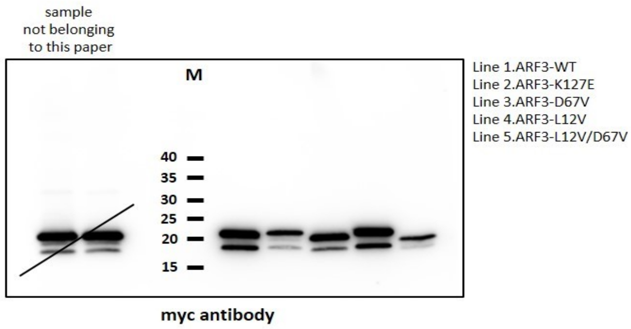

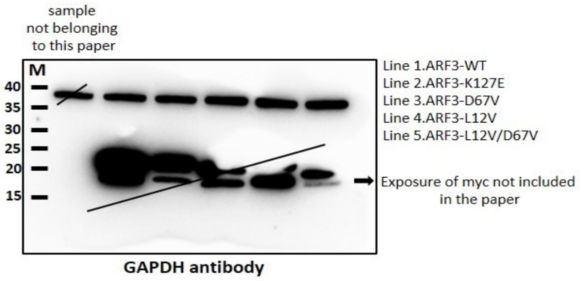

Raw uncropped blots relative to Figure S4a: Raw western blots from COS-1 cells showing protein expression of myc-tagged ARF3WT and mutants (K127E, D67V, L12V and L12VD67V, ~23kDa) normalized to reference protein (GAPDH, ~36kDa). The lanes crossed with oblique lines contain samples not belonging to this study (upper left and lower leftt) or an exposure not included in the paper (lower).

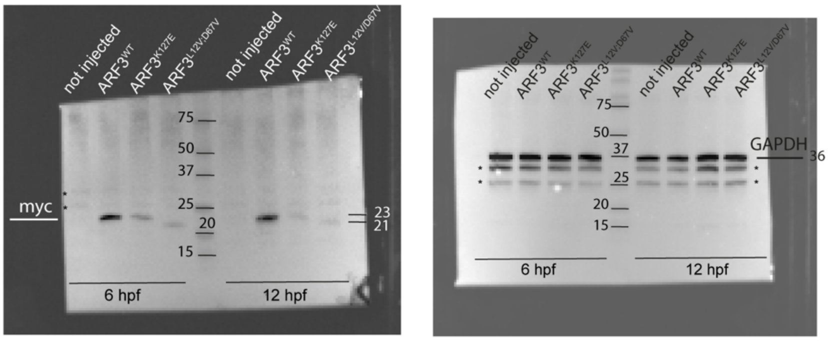

Raw uncropped blots relative to Figure S4b: Uncropped raw western blots showing protein expression of not injected, myc-tagged ARF3WT and mutants (K127E, L12VD67V, ~23kDa) at 6 and 12 hpf normalized to reference protein (GAPDH, ~36kDa). Precision Plus Dual Color Standard (Biorad, 1610374).

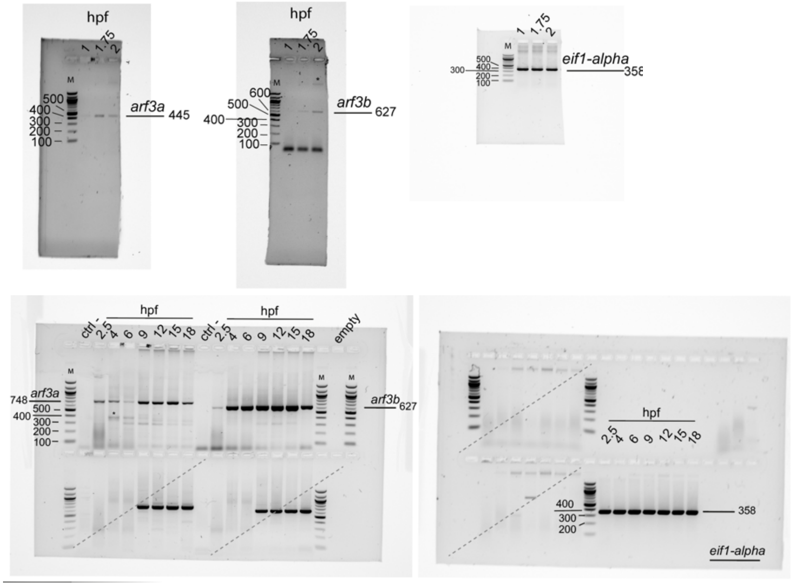

Raw uncropped gels relative to Figure S9: Uncropped raw agarose gels showing gene expression of endogenous arf3a and arf3b paralogs in zebrafish obtained by RT-PCR from total RNA isolated from embryos (from 2 to 18 hpf). The wells with dotted lines contain samples not belonging to this study. Quick Load Purple 100bp DNA ladder (NEB, N0551S)

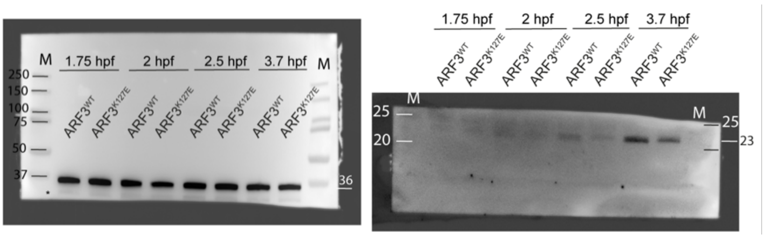

Raw uncropped blots relative to Figure S14: Uncropped raw western blot showing protein expression of myc-tagged ARF3 mutants (WT, K127E, 23 kDa) in zebrafish embryos at early embryogenesis (1.5 - 3.7 hpf) normalized to reference protein (GAPDH, 37 kDa). Precision Plus Dual Color Standard (Biorad, 1610374).
