# Supplementary material for: Dominant ARF3 variants disrupt Golgi integrity and cause a neurodevelopmental disorder recapitulated in zebrafish
Source: Nat Commun. 2022 Nov 11;13:6841. doi: 10.1038/s41467-022-34354-x (PMC9652361; doi:10.1038/s41467-022-34354-x)
Supplement: Supplementary file 3 — Description of Additional Supplementary Files [file 41467_2022_34354_MOESM3_ESM.pdf]

Description of Additional Supplementary Files

File Name: Supplementary Movie 1

Description: Time-lapse imaging of GA dynamics in COS-1 cells transfected with mCherry-tagged ARF3WT and ARF3K127E (magenta) and EGFP-GalT (green). Consecutive confocal acquisitions were performed with a time interval of 15 minutes over 2 hours. Scale bar: 20 μm
