# Supplementary material for: Dominant ARF3 variants disrupt Golgi integrity and cause a neurodevelopmental disorder recapitulated in zebrafish
Source: Nat Commun. 2022 Nov 11;13:6841. doi: 10.1038/s41467-022-34354-x (PMC9652361; doi:10.1038/s41467-022-34354-x)
Supplement: Supplementary file 5 — Reporting Summary [file 41467_2022_34354_MOESM5_ESM.pdf]

Corresponding author(s): Antonella Lauri, Marco Tartaglia

Last updated by author(s): 26.09.022

Reporting Summary

Nature Portfolio wishes to improve the reproducibility of the work that we publish. This form provides structure for consistency and transparency in reporting. For further information on Nature Portfolio policies, see our Editorial Policies and the Editorial Policy Checklist.

Statistics

For all statistical analyses, confirm that the following items are present in the figure legend, table legend, main text, or Methods section.

n/a	Confirmed	
☐	☒	The exact sample size (*n*) for each experimental group/condition, given as a discrete number and unit of measurement
☐	☒	A statement on whether measurements were taken from distinct samples or whether the same sample was measured repeatedly
☐	☒	The statistical test(s) used AND whether they are one- or two-sided<br>*Only common tests should be described solely by name; describe more complex techniques in the Methods section.*
☒	☐	A description of all covariates tested
☐	☒	A description of any assumptions or corrections, such as tests of normality and adjustment for multiple comparisons
☐	☒	A full description of the statistical parameters including central tendency (e.g. means) or other basic estimates (e.g. regression coefficient) AND variation (e.g. standard deviation) or associated estimates of uncertainty (e.g. confidence intervals)
☐	☒	For null hypothesis testing, the test statistic (e.g. *F*, *t*, *r*) with confidence intervals, effect sizes, degrees of freedom and *P* value noted<br>*Give P values as exact values whenever suitable.*
☒	☐	For Bayesian analysis, information on the choice of priors and Markov chain Monte Carlo settings
☒	☐	For hierarchical and complex designs, identification of the appropriate level for tests and full reporting of outcomes
☒	☐	Estimates of effect sizes (e.g. Cohen's *d*, Pearson's *r*), indicating how they were calculated

Our web collection on statistics for biologists contains articles on many of the points above.

Software and code

Policy information about availability of computer code

Data collection	Sequencing and WES analysis: Target enrichment kits, sequencing platforms, data analysis, and whole exome sequencing (WES) statistics are reported in Supplementary Table 3-7 and in the Supplementary Methods of the present manuscript. Software used for data collection: Illumina Experiment v.1.19.1, Illumina BaseSpace Hub v.5.43 for fastq file generation (https://help.basespace.illumina.com/overview/software-overview)<br>TEM acquisition software: iTEM3 software (Olympus)<br>Microscopy image acquisitions: images were acquired using Leica or Olympus microscopes equipped with; LASX v.3.0, 3.5, 3.7 and 4.5 (Leica), FV10-ASW v.4.1 and cellSens Standard software v.1.14 (Olympus)<br>Immunoblots and gel staining were acquired with Alliance Q9 Mini 18.02-SN software (Uvitec)
Data analysis	WES data analysis: WES data processing, read alignment to the GRCh37/hg19 version of genome assembly, and variant filtering and prioritization by allele frequency, predicted functional impact, and inheritance models were performed as previously reported by participant units. The methods, instruments and softwares adopted by all the centres which contributed to this study are reported in the Supplementary method section. The software and tools used for WES data processing and variants functional annotation are open source and publicly available: Illumina CASAVA 1.8.2<br>VarSeq software (Golden Helix, Inc v1.4.6) (https://www.goldenhelix.com/products/VarSeq/index.html),<br>BWA-MEM v.2 (https://github.com/bwa-mem2/bwa-mem2),<br>HaplotypeCaller (GATK v3.8) https://github.com/lh3/bwa, https://github.com/broadinstitute/gatk,<br>SnpEff v.5.0 https://github.com/pcingola/SnpEff,<br>dbNSFP v.4.1 and 4.2 https://sites.google.com/site/jpopgen/dbNSFP,<br>Combined Annotation Dependent Depletion (CADD) v.1.4 and 1.64 https://github.com/kircherlab/CADD-scripts,

Mendelian Clinically Applicable Pathogenicity (M-CAP) v.1.0,
Intervar v.2.0.1 (http://wintervar.wglab.org),
GERP++,
Genomics Viewer (IGV v2.4),
Metadome v1.0.1(stuart.radboudumc.nl/metadome),
Human Gene Mutation Database Professional (HGMD, Release 2020.2).

Rate constants calculation and graphs generation: GraFit v.5.0.13, GraphPad v.9 (Prism).

Multiple alignment: MUSCLE v.5 (EBI) https://www.ebi.ac.uk/Tools/msa/muscle/

Structure inspection and analysis: VMD visualization software v.1.9.3, GROMACS 2020.2., UCSF Chimera v.1.15

Microscopy images analysis: IMARIS software v.9.5 (Bitplane), LASX (Leica v.3.0, 3.3, 3.7 and 4.5), Fiji (Image J), FV10-ASW v.4.1 and Olympus cellSens Standard software v.1.14. HyVolution v.2 (Leica Microsystems)
Statistical analysis: GraphPad v.9 (Prism)., Oriana 4.0

For manuscripts utilizing custom algorithms or software that are central to the research but not yet described in published literature, software must be made available to editors and reviewers. We strongly encourage code deposition in a community repository (e.g. GitHub). See the Nature Portfolio guidelines for submitting code & software for further information.

Data

Policy information about availability of data

All manuscripts must include a data availability statement. This statement should provide the following information, where applicable:

- Accession codes, unique identifiers, or web links for publicly available datasets
- A description of any restrictions on data availability
- For clinical datasets or third party data, please ensure that the statement adheres to our policy

The clinical data were collected after signed consent forms. The entire dataset is included within the manuscript. Given the progressive nature of the disease, additional information eventually collected after this publication will be made available upon request to the corresponding authors (antonella.lauri@opbg.net, marco.tartaglia@opbg.net) and referring clinicians. The sequencing data are available under restricted access for privacy/ethical reasons, access can be obtained contacting the corresponding authors. The ARF3 variants identified in this study have been deposited in the ClinVar database under the following accession codes: SCV002549683 (c.34C>G, p.Leu12Val) [https://www.ncbi.nlm.nih.gov/clinvar/variation/1697208/?oq=SCV002549683&m=NM_001659.3(ARF3):c.34C%3EG%20(p.Leu12Val)], SCV002549684 (c.95C>A, p.Thr32Asn) [https://www.ncbi.nlm.nih.gov/clinvar/variation/1697209/?oq=SCV002549684&m=NM_001659.3(ARF3):c.95C%3EA%20(p.Thr32Asn)], SCV002549685 (c.139C>T, p.Pro47Ser) [https://www.ncbi.nlm.nih.gov/clinvar/variation/1697210/?oq=SCV002549685&m=NM_001659.3(ARF3):c.139C%3ET%20(p.Pro47Ser)], SCV002549686 (c.200A>T, p.Asp67Val) [https://www.ncbi.nlm.nih.gov/clinvar/variation/1697211/?oq=SCV002549686&m=NM_001659.3(ARF3):c.200A%3ET%20(p.Asp67Val)], SCV002549687 (c.277G>A, p.Asp93Asn) [https://www.ncbi.nlm.nih.gov/clinvar/variation/1697212/?oq=SCV002549687&m=NM_001659.3(ARF3):c.277G%3EA%20(p.Asp93Asn)], and SCV002549688 (c.379A>G, p.Lys127Glu) [https://www.ncbi.nlm.nih.gov/clinvar/variation/1697213/?oq=SCV002549688&m=NM_001659.3(ARF3):c.379A%3EG%20(p.Lys127Glu)]. The UCSC GRCh37/hg19 human genome assembly used as reference for reads alignment is available at https://www.ensembl.org/info/website/tutorials/grch37.html. The dbSNP150, gnomAD V.2.1.1, ClinVar and COSMIC v.96 databases used in this paper are available at https://gnomad.broadinstitute.org/, https://genome.ucsc.edu/cgi-bin/hgTrackUi?db=hg38&g=snp150Common and (https://www.ncbi.nlm.nih.gov/clinvar/), https://cancer.sanger.ac.uk/cosmic, respectively. The raw blots and raw data for the different measures of this study are provided in the Supplementary Information/Source data file. Source data are provided with this paper. Due to the large size of each dataset, all the raw imaging data, supporting the findings of the work are available from the corresponding authors upon request. All the constructs generated in this study will be shared upon request to the corresponding authors.

Human research participants

Policy information about studies involving human research participants and Sex and Gender in Research.

Reporting on sex and gender	Sex and gender are carefully reported in the clinical reports and table for medical scope only. Patients' sex and gender is self-reported and confirmed by genomic analysis. Sex- and gender-based analysis has not been performed because it goes beyond the scope of the study.
Population characteristics	Age, genotypic information, and clinical findings are reported in the clinical reports and table to describe the disease's clinical variability and natural history. Due to the aim of the study and the sample size, no specific recruiting and cohort design policy has been adopted. The recruitment has been arranged based on the molecular findings (see below).
Recruitment	Patient 1 was enrolled in the "Undiagnosed Patient Program" at Ospedale Pediatrico Bambino Gesù (Rome, Italy). The program is dedicated to subjects with complex clinical phenotypes molecularly unexplained.<br>All the other patients were recruited via GeneMatcher (https://genematcher.org/). No recruitment in the study was made after the submission of the orginal manuscript.
Ethics oversight	The use of clinical data and biological material (clinical data collection, biological material collection, genomic and functional studies) has been approved by the Ospedale Pediatrico Bambino Gesù Ethical Committee, Rome (Prot: 1719_OPBG_2018, December 2018). The other subjects were referred for diagnostic genetic testing. Clinical data and DNA samples were collected, stored and used following procedures in accordance with the ethical standards of the declaration of Helsinki

protocols, and after signed consents from the participating families. Permission was obtained to publish photographs of patients

Note that full information on the approval of the study protocol must also be provided in the manuscript.

Field-specific reporting

Please select the one below that is the best fit for your research. If you are not sure, read the appropriate sections before making your selection.

☒ Life sciences ☐ Behavioural & social sciences ☐ Ecological, evolutionary & environmental sciences

For a reference copy of the document with all sections, see nature.com/documents/nr-reporting-summary-flat.pdf

Life sciences study design

All studies must disclose on these points even when the disclosure is negative.

Sample size	Patients'data: sample size calculation was not performed as it does not apply to this study as the size of the cohort was constrained by the rarity of the disorder. The patients reported in the study were all the available subjects who were identified through matchmaking at the time of first submission. Patients identified from GeneMatcher matches after the submission of the original submission were not enrolled in the study.<br>Functional validation:statistical calculation of the sample size for the in vitro experiments was not performed. The size of the sample was chosen based following the common pratcise in cell biology. Calculation of the sample size for zebrafish embryos experiments was performed based on the effect size derived by the clinical phenotype in patients and using power analysis (ANOVA) function with alpha-error = 0.05 and beta-error = 0.2 within the G-power software.<br>Given the multiple parameters analyzed for each phenotype (brain, axis etc), and the use of both in vitro and in vivo samples, the sample size was sufficient to support pathogenicity of the mutations found at different levels.
Data exclusions	Patients'data: no molecular nor clinical data were deliberately excluded.<br>Funcitonal validation: data exclusion was carried out if samples (cells or embryos) were physically damaged.
Replication	The number of independent replicates from which the analysis is derived is indicated in each figure legend and summarized here.<br><br>In vitro experiments:<br>-ARF3 expression,stability and activity: three independent replicates;<br>-Nucleotide exchange and GTP hydrolysis: two independent replicates;<br><br>-Golgi morphological analysis in COS-1: 3 independent replicates;<br><br>-Live Golgi fragmentation in ARF3K127E expressing cells (time lapse in COS-1 cells): a single replicate;<br>-Cop-I assembly: 18 (WT, T32N); 26 (K127E); 16 (L12V/D67V) and 15 (P47S and D93N) cells from a single replicate;<br><br>-Tnf clustering: 3 independent replicates (WT) and 2 independent replicates for all the other ARF3 mutants;<br><br>-TEM Golgi imaging: 2 independent replicates;<br><br>-Rab5 /Tnf colocalization: 13 (WT,K127E,T32N), 16 (L12V/D67V, D93N) and 21 (P47S) cells from a single replicate;<br><br>- Rab11/Tnf colocalization: 10 (WT), 6 (K127E), 16 (L12V/D67V), 10 (P47S), 9 (D93N) and 7 (T32N) cells from a single replicate;<br><br>-Lamp2/Tnf colocalization: 8 cells for set 1 from one replicate, 10 (WT) and 9 (T32N) for ser 2 from one replicate<br><br>- Fluorescence intensity analysis of ARF3mCherry expression in COS-1 cells: 88 (WT), 100 (L12V/D67V) and 149 (D93N) cells from a single replicate;<br><br>In vivo experiments:<br>- Golgi fragmentation on zebrafish gastrula cells: 4 (WT) and 8 (K127) embryos in total from two independent batches, 1 (Q71L), 2 (T31N) and 3 (D93N) embryos (replicates) from one batch;<br><br>-ARF3 expression (protein) in vivo (late gastrula and segmentation): one batch (replicate);<br><br>-ARF3 expression (protein) in vivo (early embryos -maternal and zygotic expression): 2 independent batches (replicates);<br><br>-arf3a/b expression profile (RNA) in vivo: one batch (replicate);<br><br>-survival rate in fish expressing WT and mutant ARF3: one replicate (embryos from pooled batches);<br><br>-phenotyping in fish expressing WT and mutant ARF3: 4 (not inj, WT), 3(D93N) and 2 (K127E, L12V/D67V, P47S, T32N) independent batches (replicates);

-phenotyping in fish injected with arf3a/b morpholino : one batch (replicate);

-arf3a/b morpholino rescue experiment (co-injection of WT and mutant ARF3 +/- arf3a/b MO) : 3 (D93N) and 2 (other mutations) batches (replicates).

- microcephaly assessment 48hpf: 25 (not injected), 23 (WT); 22 (K127E) embryos of one batch for set 1 and 29 (WT) 25 (L12V/D67V); 28 (P47S); 30 (D93N) and 29 (T32N) embryos of one batch for set 2.

-microcephaly assessment 4.5dpf: 30 (not injected, WT, D93N); 27 (P47S); and 28 (T32N) embryos from one batch.

-brain volume assessment in live embryos 48hpf: 4 (WT, K127E) embryos from one batch for set 1 and 3 (WT, L12V/D67V) and 4 (P47S and D93N) embryos from one batch for set 2.

- brain volume assessment in fixed specimens at 48hpf : 5 (WT) and 6 (K127E) embryos from one batch

-white matter assessment: set 1 : 8 (WT), 7 (K127E), 3 (L12V/D67V) and 5 (D93N) embryos from one batch; set 2: 5 (WT), 5 (P47S), 6 (T32N) embryos from one batch;

-assessment of proliferative cells and cell cycle within the forebrain of fish mutants: 5 embryos from one batch;

-assessment of cell death within the forebrain of fish mutants: 6 (WT, L12V/D67V, P47S); 5 (K127E) embryos from one batch;

-Spindle morphology in zebrafish forebrain: set 1: 28 (WT), 18 (K127) cells from one embryo each, set 2: 12 (WT) and 19 (D93N) cells from one embryo each

-trunk/notochord assessment in embryos: set 1 (not inj., WT, K127E, L12V/D67V): three independent batches (replicates); set 2 (WT, P47S, D93N, T32N): one batch (replicate);
-number of notochord curvatures per embryos: set 1: 9 (not inj), 17 (WT), 15 (K127E) and 11 (L12VD67V) embryos from three batches; set 2: 13 (WT), 28 (P47S), 19 (D93N), 24 (T32N) embryos from one batch.

- in situ hybridization of MyoD and Krox20, AP extension : set 1: 10 (WT, K127E, L12VD67V) from one batch; set 2: 18 (WT), 22 (P47S), 24 (D93N), 16 (T32N) embryos from one batch
-CE index: one batch (replicate).

-number of somites : 24 (not inj), 18 (WT), 13 (K127E), 13 (L12V/D67V), 22 (P47S), 24 (D93N) and 16 (T32N) embryos from one batch;

-angle measurament (13hpf): set 1 : 23 (not inj), 24 (WT), 27 (K127E), 27 (D93N) embryos from one batch; set 2: 21 (WT), 21 (L12V/D67V), 21 (P47S), and 24 (T32N) embryos from one batch;

- cell protrusion during gastrulation in embryos: 10 cells from one embryo.

When retrieving statistical information from biological replicates (cells, embryos or batches) mean (or median) and SEM or SD (or interquartile range, min and max) are reported. A statement on the "n" indicating the number of cells/embryos in each experiment used is also indicated in the figure legend

Randomization

WT and mutant samples were produced from the same original population (batch) of cells/embryos. Allocation of cells and embryos to the WT or mutant sample was randomly assigned. Randomization was followed with respect to imaging sequence. Randomization does not apply to the cohort of patients included in the study.

Blinding

Experiments were not performed blindly as severe or major features associated with the mutations (such as microcephaly or low fluorescent signal of the protein expression) where clearly identifiable in the populations studied. Key results were assessed by at least two researchers independently.

Reporting for specific materials, systems and methods

We require information from authors about some types of materials, experimental systems and methods used in many studies. Here, indicate whether each material, system or method listed is relevant to your study. If you are not sure if a list item applies to your research, read the appropriate section before selecting a response.

Materials & experimental systems

n/a	Involved in the study	
☐	☒	Antibodies
☐	☒	Eukaryotic cell lines
☒	☐	Palaeontology and archaeology
☐	☒	Animals and other organisms
☒	☐	Clinical data
☒	☐	Dual use research of concern

Methods

n/a	Involved in the study	
☒	☐	ChIP-seq
☒	☐	Flow cytometry
☒	☐	MRI-based neuroimaging

Antibodies

Antibodies used

Primary antibodies:
mouse monoclonal anti-myc (1:1000, Cell Signaling, 2276S),
mouse monoclonal anti-β-tubulin (1:1000, Thermo Fisher, 32-2600),
mouse monoclonal anti-GAPDH (1:1000, Santa Cruz, sc-32233),
mouse monoclonal anti-Golgin 97 (1:50, Invitrogen, A21270),
rabbit polyclonal anti-b-COP (1:2000, abcam, ab2899),
rabbit monoclonal anti-Rab5 (1:200, Cell Signaling, 3547S),
mouse monoclonal anti-LAMP-2 (1:200, Santa Cruz, sc-18822),
rabbit polyclonal anti-GAPDH (1:1000, Genetex, Cat.N° GTX124503),
rabbit polyclonal anti-Elavl3+4 (1:100, GeneTex, GTX128365),
rabbit polyclonal anti-PCNA (1:250, GeneTex, GTX12449),
mouse monoclonal anti-pH3 (1:250, Abcam, ab14955),
mouse monoclonal anti-acetylated tubulin (1:250, Sigma Aldrich, T7451)

Secondary antibodies:
HRP-conjugated polyclonal anti-mouse (1:3000, Thermo Fisher, 31450),
HRP-conjugated polyclonal anti-rabbit (1:3000, Thermo Fisher, 31460)
Alexa Fluor 488 polyclonal goat anti-mouse (1:200, Invitrogen, A11017),
Alexa Fluor 633 polyclonal goat anti-mouse (1:200, Invitrogen, A21050),
Alexa Fluor 488 polyclonal goat anti-rabbit (1:200, Invitrogen, A11070),
DyLight 594 polyclonal goat anti-rabbit (1:1000, GeneTex, GTX213110-05)

Validation

All the primary antibodies were validated based on expected subcellular localization or molecular weight of the detected protein, in addition to available validation.
Mouse anti-myc (Cell Signaling 2276S): Suitable for WB, IP, IHC-P, IF-IC, FC-FP, ChIP; Reacts with all Species Expected. Citation: Napoli M, et al. ΔNp63 regulates a common landscape of enhancer associated genes in non-small cell lung cancer. Nat Commun. 2022;13(1):614. doi: 10.1038/s41467-022-28202-1. Mahuzier A. et al., Dishevelled stabilization by the ciliopathy protein Rpgrip1l is essential for planar cell polarity. J Cell Biol. 2012 Sep 3;198(5):927-40. doi: 10.1083/jcb.201111009.
Mouse anti-β-tubulin (Thermo Fisher 32-2600): Suitable for WB- IP- IHC- IF-Flow; Reacts with C. elegans, Dog, Human, Mouse, Non-human primate, Pig, Rat. Citation: Daniloski Z, et ak. Identification of Required Host Factors for SARS-CoV-2 Infection in Human Cells. Cell 2021;184(1):92-105.e16. doi: 10.1016/j.cell.2020.10.030.
Mouse anti-GAPDH (Santa Cruz sc-32233): Suitable for WB-IF-IP; Reacts with mouse, rat, human, rabbit, Xenopus laevis. Citation: Wang, Y, et al. Precise tumor immune rewiring via synthetic CRISPRa circuits gated by concurrent gain/loss of transcription factors. Nat Commun. 2022;13(1):1454. doi: 10.1038/s41467-022-29120-y
Mouse anti-Golgin 97 (Invitrogen A21270): Suitable for WB- IP- IHC- IF-Flow-IP-IM; Reacts with dog, human, mouse, non-human primate. Advanced Verification from the company:This Antibody was verified by Knockdown to ensure that the antibody binds to the antigen stated. Citation: Low BSJ, et al.Decreased GLUT2 and glucose uptake contribute to insulin secretion defects in MODY3/HNF1A hiPSC-derived mutant β cells. Nat Commun. 2021;12(1):3133. doi: 10.1038/s41467-021-22843-4.
Rabbit anti-b-COP (abcam ab2899): Suitable for WB- IP- IF; Reacts with mouse, rat, hamster, cow, human, non human primates. Citation: Sasako T, et al. Hepatic Sdf2l1 controls feeding-induced ER stress and regulates metabolism. Nat Commun. 2019;10(1):947. doi: 10.1038/s41467-019-08591-6.
Rabbit anti-Rab5 (Cell Signaling 3547S): Suitable for WB-IF; Reacts with human, mouse, rat, monkey. Citation: David Porciani, et. al. Modular cell-internalizing aptamer nanostructure enables targeted delivery of large functional RNAs in cancer cell lines. Nat Commun. 2018;9(1):2283. doi: 10.1038/s41467-018-04691-x.
Mouse Anti-LAMP-2 (Santa Cruz, sc-18822): Suitable for WB-IP-IF-IHC-Flow; Reacts with human. Citation: Kim K, et al. O-GlcNAc modification of leucyl-tRNA synthetase 1 integrates leucine and glucose availability to regulate mTORC1 and the metabolic fate of leucine. Nat Commun. 2022;13(1):2904. doi: 10.1038/s41467-022-30696-8.
Rabbit anti-Elavl 3+4 (Genetex, Cat.N° GTX128365): Suitable for ICC/IF, IHC-Fr, IHC-Wm. Reacts with human and zebrafish. Citation: Berg EM, Bertuzzi M, Ampatzis K. Complementary expression of calcium binding proteins delineates the functional organization of the locomotor network. Brain Struct Funct. 2018 Jun;223(5):2181-2196. doi: 10.1007/s00429-018-1622-4.
Rabbit anti-PCNA (Genetex, Cat.N° GTX12449): Suitable for WB, IHC-P, IHC-Fr, IHC-Wm, Immunostaining. Reacts with zebrafish, japanese medaka, nothobranchius furzeri. Citation: Than-Trong E et al. Neural stem cell quiescence and stemness are molecularly distinct outputs of the Notch3 signalling cascade in the vertebrate adult brain. Development. 2018 May 15;145(10):dev161034. doi: 10.1242/dev.161034.
Mouse anti-pH3 (Abcam, Cat.N° ab14955): Suitable for IHC-P, ELISA, ICC, Flow Cyt, WB. Reacts with Mouse, Human, Drosophila melanogaster, Recombinant fragment. Predicted to work with: Rat, Chicken, Saccharomyces cerevisiae, Xenopus laevis, Arabidopsis thaliana, Caenorhabditis elegans, Indian muntjac, Monkey, Schizosaccharomyces pombe, Zebrafish, Mammals, Tobacco, Chlamydomonas reinhardtii, African green monkey, Aspergillus nidulans, Neurospora crassa, Oncopeltus. Citation: Seredick S. et al., Lhx3 and Lhx4 suppress Kolmer-Agduhr interneuron characteristics within zebrafish axial motoneurons. Development. 2014 Oct;141(20):3900-9. doi: 10.1242/dev.105718.
Rabbit polyclonal anti-GAPDH (Genetex, Cat.N° GTX124503): Suitable for WB. Reacts with human and zebrafish. Citation: Mikdache A. et al., Rgs4 is a regulator of mTOR activity required for motoneuron axon outgrowth and neuronal development in zebrafish. Sci Rep. 2021 Jun 25;11(1):13338. doi: 10.1038/s41598-021-92758-z.
Mouse anti-acetylated tubulin (Sigma Aldrich, T7451): Suitable for DB, EM, IHC, RIA, WB. Reacts with plant, hamster, rat, mouse, human, pig, monkey, frog, invertebrates, bovine, protista, chicken. Citation: Rieke J.M. et al., SLC20A1 Is Involved in Urinary Tract and Urorectal Development. Front. Cell Dev. Biol. August 2020. Vol.8. doi:10.3389/fcell.2020.00567.

Eukaryotic cell lines

Policy information about cell lines and Sex and Gender in Research

Cell line source(s)	COS1 from ATCC (https://www.atcc.org/products/crl-1650) were kindly provided by collegues at ISS (Istituto Superiore di Sanità), Italy
Authentication	no authentication performed
Mycoplasma contamination	Mycoplasma test were performed weekly and tested negative
Commonly misidentified lines (See ICLAC register)	no commonly misidentified cell lines were used in the study

Animals and other research organisms

Policy information about studies involving animals; ARRIVE guidelines recommended for reporting animal research, and Sex and Gender in Research

Laboratory animals	Zebrafish (Danio rerio) is used in the study, WT strain and transgenic lines used (NHGRI, Tg(Xla.Tubb:DsRed) and Tg(XlEef1a1:dclk2DeltaK-GFP)) are also reported in the "Methods"section. Embryos < 5dpf were involved in the study.
Wild animals	the study did not involve wild animals.
Reporting on sex	Information of sex was not collected as embryos do not show dimorphism
Field-collected samples	the study did not involve field-collected samples
Ethics oversight	All animal experiments were conducted under the approval of the Italian Ministry of Health (DGSA -Direzione generale della sanità animale e dei farmaci veterinari, 23/2019-PR).

Note that full information on the approval of the study protocol must also be provided in the manuscript.
